# The effects of exposure to solar radiation on human health

**DOI:** 10.1007/s43630-023-00375-8

**Published:** 2023-03-01

**Authors:** R. E. Neale, R. M. Lucas, S. N. Byrne, L. Hollestein, L. E. Rhodes, S. Yazar, A. R. Young, M. Berwick, R. A. Ireland, C. M. Olsen

**Affiliations:** 1 Population Health Program, QIMR Berghofer Medical Research Institute, Brisbane, QLD Australia; 2 School of Public Health, University of Queensland, Brisbane, QLD Australia; 3 National Centre for Epidemiology and Population Health, Australian National University, Canberra, ACT Australia; 4 School of Medical Science, Faculty of Medicine and Health, University of Sydney, Sydney, NSW Australia; 5 Erasmus MC Cancer Institute, Rotterdam, The Netherlands; 6 Netherlands Comprehensive Cancer Organisation, Utrecht, The Netherlands; 7 Dermatology Research Centre, School of Biological Sciences, University of Manchester, Salford Royal Hospital, Northern Care Alliance NHS Trust, Manchester, UK; 8 Garvan Medical Research Institute, Sydney, NSW Australia; 9 Kings College London, London, UK; 10 University of New Mexico Comprehensive Cancer Center, Albuquerque, USA; 11 Frazer Institute, University of Queensland, Brisbane, QLD Australia

## Abstract

This assessment by the Environmental Effects Assessment Panel (EEAP) of the Montreal Protocol under the United Nations Environment Programme (UNEP) evaluates the effects of ultraviolet (UV) radiation on human health within the context of the Montreal Protocol and its Amendments. We assess work published since our last comprehensive assessment in 2018. Over the last four years gains have been made in knowledge of the links between sun exposure and health outcomes, mechanisms, and estimates of disease burden, including economic impacts. Of particular note, there is new information about the way in which exposure to UV radiation modulates the immune system, causing both harms and benefits for health. The burden of skin cancer remains high, with many lives lost to melanoma and many more people treated for keratinocyte cancer, but it has been estimated that the Montreal Protocol will prevent 11 million cases of melanoma and 432 million cases of keratinocyte cancer that would otherwise have occurred in the United States in people born between 1890 and 2100. While the incidence of skin cancer continues to rise, rates have stabilised in younger populations in some countries. Mortality has also plateaued, partly due to the use of systemic therapies for advanced disease. However, these therapies are very expensive, contributing to the extremely high economic burden of skin cancer, and emphasising the importance and comparative cost-effectiveness of prevention. Photodermatoses, inflammatory skin conditions induced by exposure to UV radiation, can have a marked detrimental impact on the quality of life of sufferers. More information is emerging about their potential link with commonly used drugs, particularly anti-hypertensives. The eyes are also harmed by over-exposure to UV radiation. The incidence of cataract and pterygium is continuing to rise, and there is now evidence of a link between intraocular melanoma and sun exposure. It has been estimated that the Montreal Protocol will prevent 63 million cases of cataract that would otherwise have occurred in the United States in people born between 1890 and 2100. Despite the clearly established harms, exposure to UV radiation also has benefits for human health. While the best recognised benefit is production of vitamin D, beneficial effects mediated by factors other than vitamin D are emerging. For both sun exposure and vitamin D, there is increasingly convincing evidence of a positive role in diseases related to immune function, including both autoimmune diseases and infection. With its influence on the intensity of UV radiation and global warming, the Montreal Protocol has, and will have, both direct and indirect effects on human health, potentially changing the balance of the risks and benefits of spending time outdoors.

**Graphical abstract:**

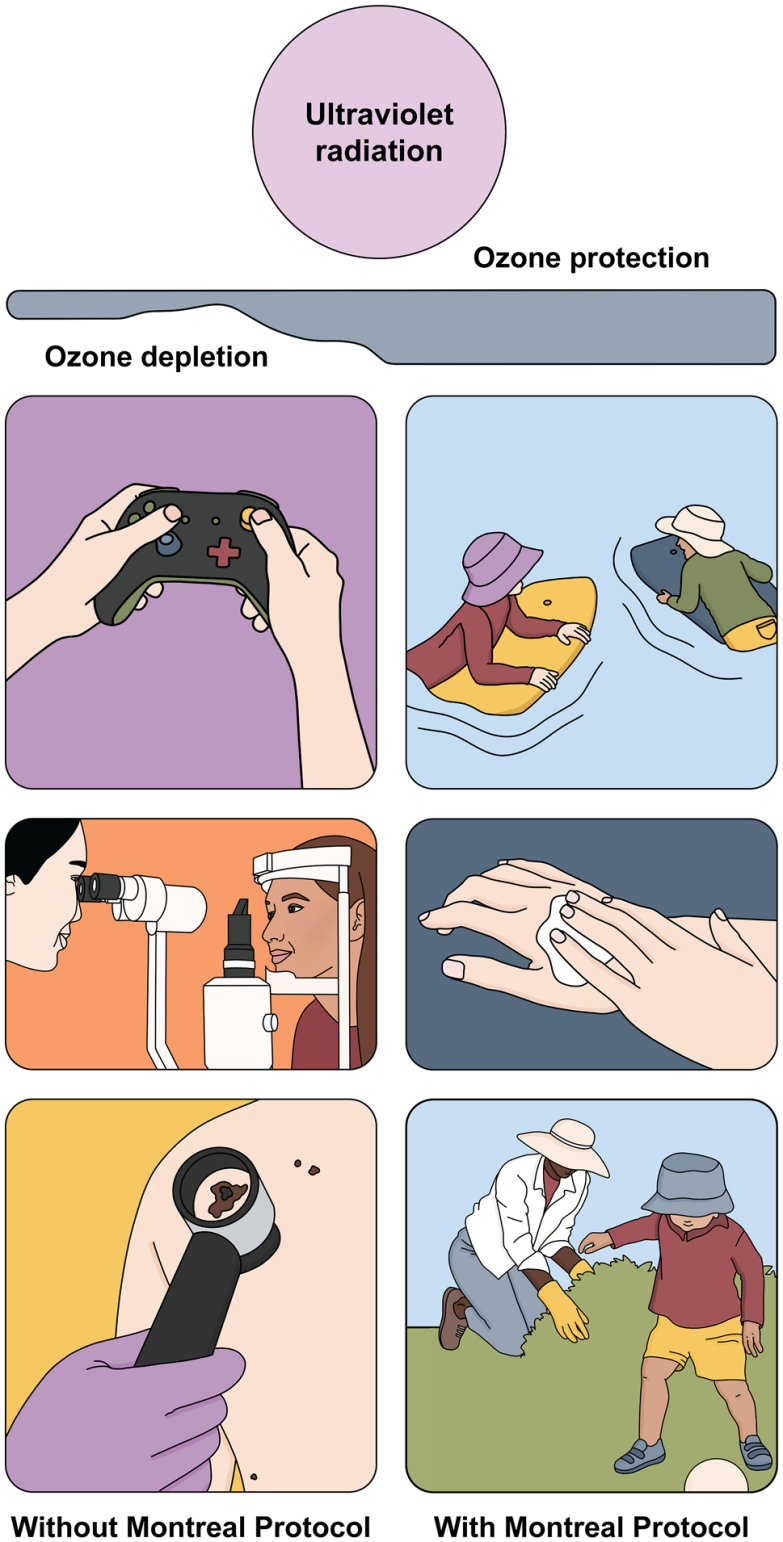

**Supplementary Information:**

The online version contains supplementary material available at 10.1007/s43630-023-00375-8.

## Introduction

The Montreal Protocol on Substances that Deplete the Ozone Layer and its Amendments (most recently Kigali in 2016) have prevented substantial depletion of stratospheric ozone and facilitated its recovery, with a marked effect on ultraviolet (UV) radiation and reduction in global warming. In the absence of the Montreal Protocol, the erythemally weighted UV irradiance, indicated by the UV Index, would have increased by up to 20% between 1996 and 2020 in the region where most of the world’s population lives (between 50°N and 50°S of the equator) [1]. With the Montreal Protocol, it is projected that UV radiation will decline at mid-latitudes over the remainder of the twenty-first century, although in urban areas where air quality is improving, UV radiation at the Earth’s surface is likely to increase. The Montreal Protocol has contributed to a reduction in global warming, as the ozone-depleting chemicals controlled under the Protocol are also potent greenhouse gases.

The changes brought about by the Montreal Protocol have important effects on human well-being, both directly and indirectly. In this assessment, we focus largely on direct effects due to human exposure to UV radiation, but human health is also influenced by air quality [2] and impacts of UV radiation on terrestrial [3] and aquatic [4] ecosystems, and materials [5]. Direct effects occur due to ozone-driven changes in the intensity of UV radiation, influencing the time outdoors before damage to the skin and eyes occurs. These changes in UV irradiance, along with climate change, influence sun exposure and sun protection behaviour. However, changes in health outcomes linked to UV radiation also need be considered within the context of broader societal influences and changes in health service use. For example, over the past several decades day-to-day occupational and recreational activities have moved predominantly indoors, but in many countries with temperate climates, annual holidays in regions with high ambient UV radiation have become common and use of sunbeds has increased. Alongside this, the sun protection factor of sunscreens has increased and the public has been educated about how to protect the skin from the sun. In developed countries, changing practices in screening and diagnosis, particularly for skin cancer, make a considerable contribution to the observed trends. It is, thus, challenging to attribute trends in human health solely to changes in ambient UV radiation. However, with the increases in UV radiation that would have occurred in the absence of the Montreal Protocol, balancing the risks and benefits of sun exposure would have presented a far greater challenge.

We present an assessment of findings regarding the effect of UV radiation on health published since our previous Quadrennial Assessment [6]. This assessment is not a systematic literature review. Rather, we conducted a broad critical assessment of the literature to identify publications containing information that may be of interest to policy makers whose remit is to make decisions about controls of ozone-depleting substances. This Perspective is part of the topical collection: Environmental effects of stratospheric ozone depletion, UV radiation, and interactions with climate change: UNEP Environmental Effects Assessment Panel, 2022 Quadrennial Assessment (10.1007/s43630-023-00374-9).

## New knowledge about mechanisms underpinning the effects of UV radiation on health

### Genes and skin cancer

**Fig. 1:**
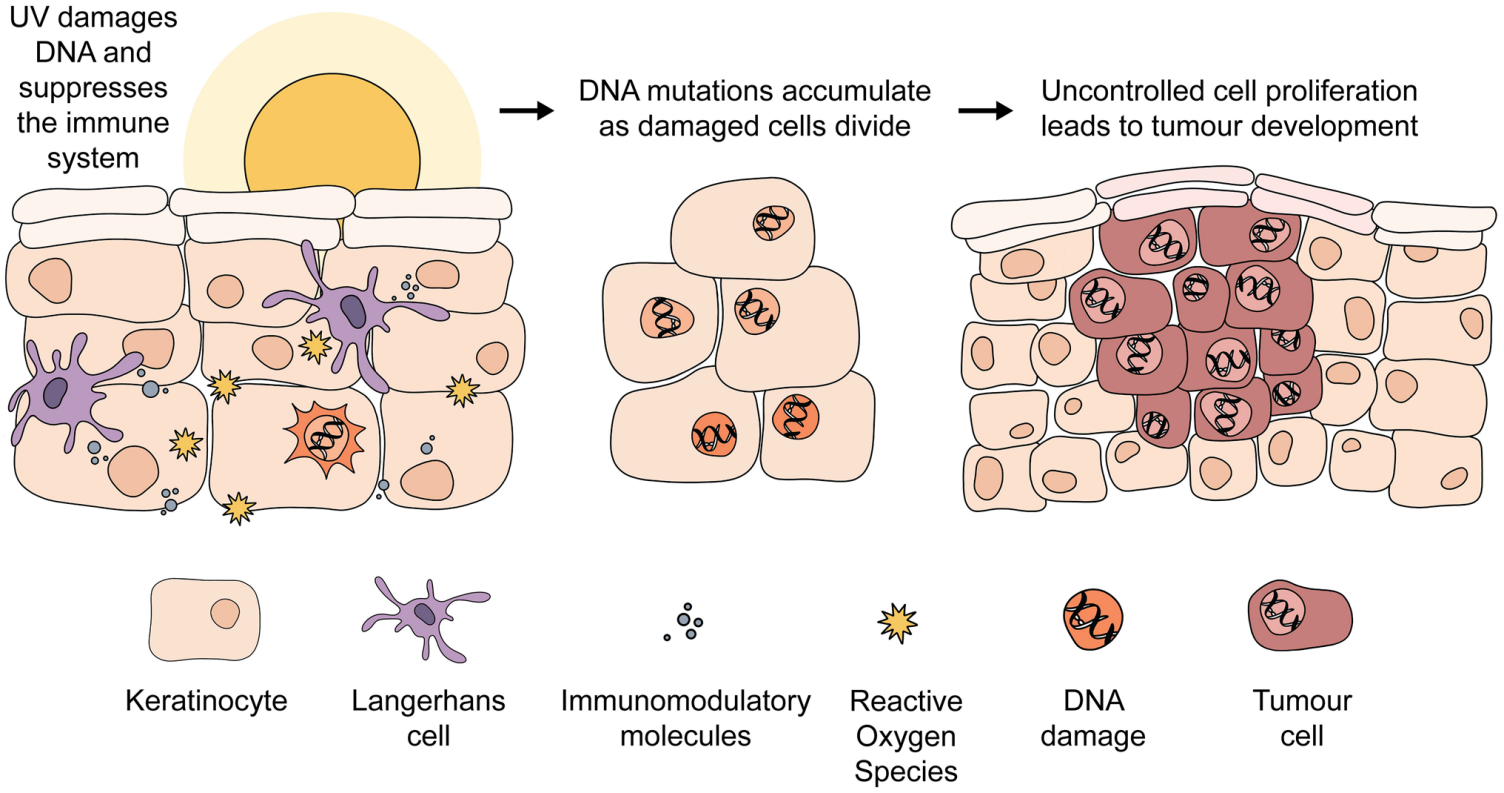
Skin cancer arises primarily as a consequence of direct and indirect (via reactive oxygen species) DNA damage and immune suppression. (Figure created by Rachael Ireland)

Skin cancer arises primarily as a consequence of UV-induced DNA damage that remains unrepaired, combined with immune suppression (Fig. 1). The past decade has seen an in-depth discovery of the genetic basis of skin cancers. Cutaneous melanomas carry distinct UV radiation mutational signatures (C > T substitutions at TpC dinucleotides (mutated base underlined), C > T substitutions at CpC and CpC dinucleotides, and high levels of T > C and T > A mutations (see Online Resource Fig. 1); the latter mutations may be caused by indirect DNA damage following exposure to UV radiation [7]. Melanocytes, from which melanomas arise, contain over 2000 genomic sites that are up to 170-fold more susceptible to UV radiation-induced damage than the average site in the genome [8]. These may serve as genetic dosimeters (i.e. indicators of UV radiation dose), which could be developed as a tool to determine risk of melanoma and, thus, the need for surveillance.

Until recently, it was believed that cyclobutane pyrimidine dimers (CPDs) could only be formed during exposure to UV radiation. New studies have shown that CPDs can be formed after UV radiation exposure has ended, with maximal expression 2–3 h post-irradiation, including in human skin in vivo [9]. These “dark CPDs” are formed by chemiexcitation, in which energy from UV radiation photons is transferred to chemical intermediates, including melanin intermediates, which then transfer energy to DNA, resulting in CPD formation. The biological significance of dark CPDs is unknown.

Many genetic loci associated with the risk of melanoma have been discovered. Additional variants have been identified through the use of multi-trait analysis of genome-wide association studies. Of note, new variants include those related to autoimmune traits; further functional analyses of these may identify new targets for chemoprevention of melanoma [10]. This method has also been used to identify new loci underpinning risk of keratinocyte cancer. Most variants affect both basal cell carcinoma (BCC) and squamous cell carcinoma (SCC) (collectively called keratinocyte cancer (KC)), demonstrating their shared susceptibility [11]. Loci in pigmentation, DNA repair and cell-cycle control, telomere length and immune response pathways have been identified.

### The role of UV radiation-induced immune modulation in the harms and benefits of sun exposure

Many of the harmful and beneficial effects of exposure to UV radiation are mediated through UV-induced effects on the immune system, both locally and systemically. Our immune system is responsible for protecting us from pathogens and destroying aberrant (potentially malignant) cells. At the same time, it must self-regulate to avoid over-reactions to pathogens, and to tolerate ‘self’ by not attacking self-antigens that could lead to autoimmune diseases. In most people, exposing the skin to UV radiation suppresses local (skin) immune processes, enabling malignant cells to escape immune control, but it also upregulates anti-microbial processes in the skin. It also suppresses aberrant immune responses systemically; i.e. in other, non-sun exposed, parts of the body. Exposure to UV radiation is, thus, ‘immune modulatory’ rather than solely ‘immune suppressive’.

### Mechanisms and consequences of UV radiation-induced modulation of immunity

Modulation of the immune system occurs through the direct or indirect activation of cells that reside within the epidermis and dermis, including epidermal keratinocytes, dendritic cells such as Langerhans’s cells, dermal lymphocytes, nerves, and mast cells [12]. Indirect pathways include UV radiation-induced changes in the action of cytokines and other mediators of the immune response, such as nitric oxide, *cis*-urocanic acid, ligands of the aryl hydrocarbon receptor, platelet-activating factor (PAF), prostaglandin E2, anti-microbial peptides, and vitamin D [13]. Some of these mediators lead to the recruitment of circulating immune cells from the blood. For example, following a sunburn (see Sect. 3.2), the skin is rapidly infiltrated by neutrophils, the most abundant leucocyte (white blood cell) in the circulation. Neutrophil infiltration peaks at ~ 24 h after exposure to an inflammatory (3 minimal erythema dose (MED)) dose of broadband UV-B radiation, returning to baseline 7–14 days later [14]. Neutrophils perform important anti-bacterial functions which, together with the induction of anti-microbial peptides, partly explains why skin infections are uncommon following exposure of the skin to UV radiation. UV-recruited neutrophils also produce anti-inflammatory cytokines such as IL-4 which leads to local immune suppression.

**Fig. 2:**
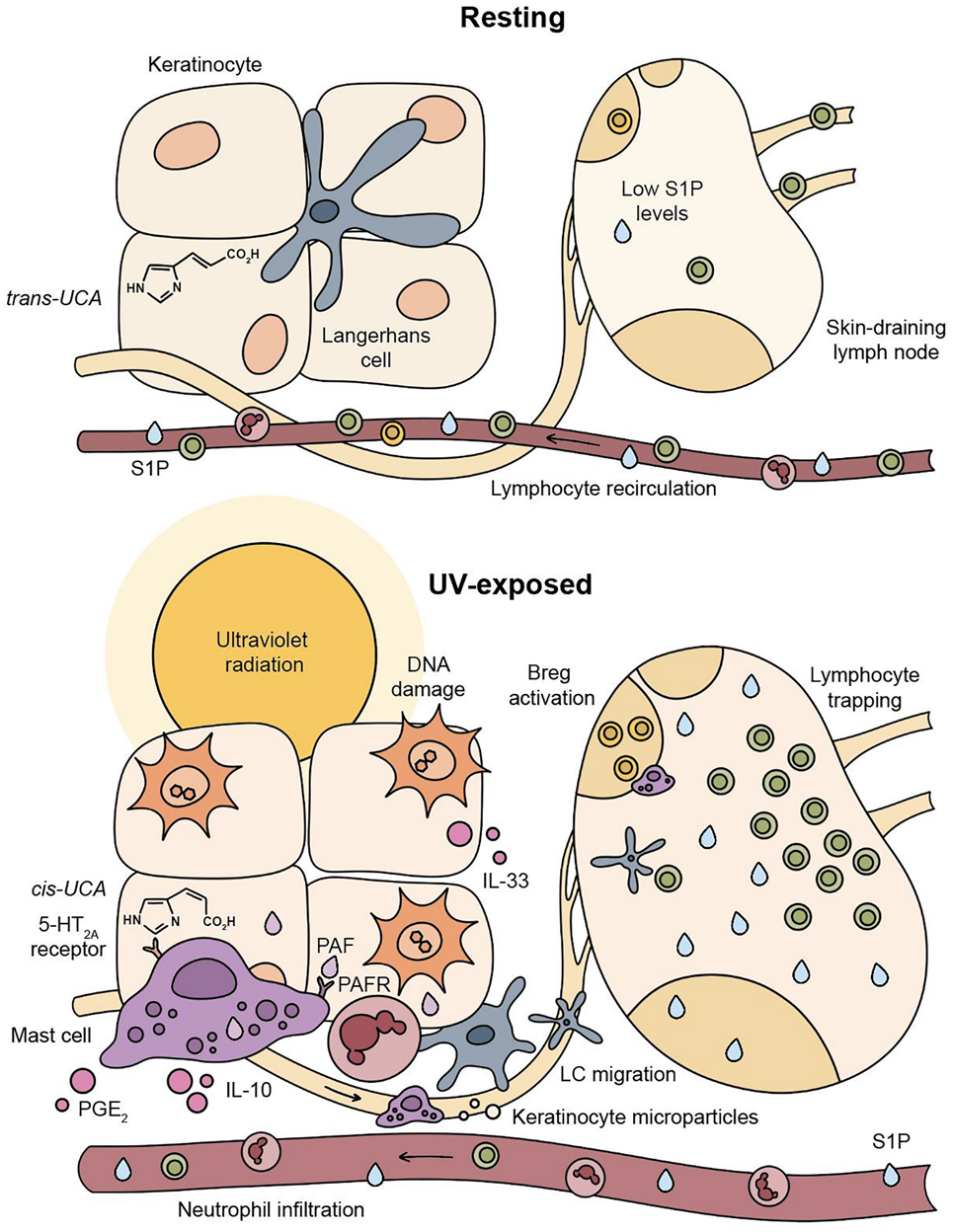
Ultraviolet radiation is immunomodulatory. The absorption of UV radiation by chromophores in the skin directly and indirectly activates cells in the epidermis and dermis, including keratinocytes, Langerhans cells (LCs), mast cells and dermal lymphocytes. Exposing the skin to UV radiation stimulates keratinocytes and mast cells to release microvesicle particles, cytokines and immunomodulatory lipids such as platelet-activating factor (PAF), which induce neutrophil and monocyte infiltration into the skin and can affect distant, non-skin cells. Skin mast cells and dendritic cells migrate into the skin-draining lymph nodes where they activate regulatory phenotypes (e.g. B_reg_). Elevated sphingosine-1-phosphate (S1P) lipid levels in the draining lymph nodes after exposure of the skin to UV radiation also contribute to systemic immune suppression by preventing lymphocyte circulation. *UCA* urocanic acid, *5-HT* 5-hydroxytryptamine, *PG* prostaglandin (Figure created by Rachael Ireland)

Dendritic cells in the skin capture, process and present antigens to other immune cells, initiating an immune response. They are versatile and ‘plastic’ in their ability to take up, process and present foreign and tumour antigens to T cells. It is this property that makes dendritic cells the ‘conductors’ of the adaptive immune response. In response to UV radiation, dendritic cells and mast cells migrate from the site of exposure to the lymph nodes that drain the skin. There, they regulate T-cell-dependent responses (reviewed in [15]) and activate immune regulatory B cells (B_Regs_—Fig. 2) [16]. Importantly, in mouse models and using solar-simulated UV radiation, blocking this UV radiation-induced migration of mast cells [17] and/or the activity of UV-activated B cells [18] prevents carcinogenesis induced by UV radiation. Other regulatory immune cells are also activated and may migrate back to UV-irradiated skin [14]. There they suppress the skin and anti-tumour immune responses, modulate inflammation, potentially enhancing wound healing [19], and/or proliferate and migrate into the circulation (reviewed in [12]). Together, these events explain why UV radiation is considered a complete carcinogen; it is able to both mutate DNA and suppress the anti-tumour immune response.

Research published since our last assessment [6] has highlighted new mechanisms by which exposing the skin to UV radiation influences immunity, including upregulation of lipids, changes in white blood cells, and alterations in the skin microbiome and transcriptome. Exposing the skin to solar-simulated UV radiation causes an increase in the production of immunomodulatory lipids such as platelet-activating factor (PAF) and PAF-like species [20]. These bioactive lipids, and changes in lipid metabolism, directly affect immune-cell phenotype and function, including increasing the production of cytokines that suppress the immune system (Fig. 2). In addition, activation of the PAF receptor in human skin induces the release of large numbers of microvesicle particles [21]. These may transport PAF and other bioactive chemicals from epidermal keratinocytes to distant immune cells and organs, thus effecting UV-B-mediated systemic immune modulation [21]. This discovery provides crucial insight into the mechanism by which exposure to UV-B radiation alters the immune system at sites that are not directly exposed to the radiation.

The effects of exposure to UV radiation on white blood cell (leukocyte) subsets in blood have been recently reviewed [13]. Exposure of mice to a single 8 kJ m^−2^ dose of solar-simulated UV radiation induces changes in the number, phenotype and function of these cells in both the innate and adaptive immune systems that typically lead to reduced activity and capacity to recirculate [22], consistent with benefits for immune-mediated disorders such as multiple sclerosis (MS) and potentially COVID-19 [23].

Several studies have identified seasonal changes in the number of leukocytes and have found the overall inflammatory milieu to be more pro-inflammatory in winter and anti-inflammatory in summer. While vitamin D is known to have effects on immune function, the effects on leukocytes were independent of vitamin D status (reviewed in [13]). In support of this work, a randomised controlled trial (RCT) of low-dose (400 IU/day) vitamin D_3_ supplementation (compared to placebo) in vitamin D-deficient (mean 25-hydroxy vitamin D [25(OH)D^1^] blood concentration = 36.1 nmol L^−1^) but otherwise healthy participants in Aberdeen, Scotland, found seasonal variation in natural T-regulatory cell populations and functions that was independent of blood 25(OH)D concentration [24].

UV irradiation of the skin causes changes in the skin microbiome [25] and transcriptome (the set of coding and non-coding RNA in cells) [26]. In people with atopic dermatitis (the most common type of eczema), 12–25 treatments over 6–8 weeks with narrowband UV-B radiation caused a shift to greater microbial diversity accompanied by reduced skin inflammation [25]. Irradiation of the skin of seven healthy male volunteers (skin type II) using solar-simulated UV radiation and doses equivalent to 0, 3 and 6 standard erythemal doses (SED) led to altered expression, mainly upregulation, of multiple genes (primarily related to DNA repair and apoptosis, immunity and inflammation, pigmentation, and vitamin D synthesis) [26]. The number of genes affected increased with increasing dose of UV radiation. UV-B (280–320 nm) and UV-A1 (340–400 nm) had similar effects on gene expression.

An abnormal cutaneous response to exposure to UV radiation may result in overactive immune responses to substances in the skin, resulting in UV-induced allergic skin conditions [27]. Evidence is also accruing to suggest that dysfunction of the skin’s innate immune system contributes to some photodermatoses, including conditions aggravated by sun exposure such as systemic lupus erythematosus (SLE) [28] and rosacea [29] (Sect. 4.3). Abnormalities of innate immunity can explain the enhanced UV-B-induced keratinocyte damage observed in cutaneous manifestations of SLE [28], and the inflammatory response to UV-B-induced keratinocyte damage in rosacea [29].

Recent studies show that irradiating the skin of mice with UV-B radiation can lead to changes in distant organs. One study demonstrated changes in gene expression in the kidney, upregulating inflammatory responses [30]. This may be one mechanism by which sun exposure in people with SLE causes acute exacerbation of nephritis (inflammation of the kidney). In another study in mice, chronic exposure of the skin to broadband UV-B radiation (100–300 m J cm^−2^ for 3 days per week for 10 weeks) significantly reduced levels of dopamine and related enzymes (tyrosine hydroxylase and dopamine beta-hydroxylase) in the blood and adrenal glands and induced marked damage in the adrenal medulla [31]. These studies add to our emerging understanding of wide-ranging systemic effects of exposing the skin to UV radiation, noting that studies in mice do not always translate to humans but that similar studies in humans may not be feasible.

## Harms of exposure to UV radiation

Human exposure to UV radiation causes harms to the skin and eyes. For the skin in particular, the risks vary according to skin pigmentation. People with deeply pigmented skin are at particularly low risk of UV-induced skin cancer, due to the type of melanin and the degree of pigmentation. In contrast, people with lightly pigmented skin are at markedly increased risk of skin cancer, particularly if they reside in areas with high ambient UV radiation. Low-dose repeated exposures to UV radiation can increase pigmentation and skin thickness, offering some protection against skin damage during subsequent exposures, a concept called habituation. However, the protection afforded is modest, with photoprotection factors (interpreted similarly to the sun protection factor (SPF) used for sunscreens) of 2–3 for people with darker skin at high northern latitudes and 10–12 for people with lighter skin types at lower European latitudes (e.g. 35°North) [32].

### Skin cancer

#### The association between exposure to UV radiation and skin cancer 

Exposing the skin to UV radiation is the primary modifiable cause of melanoma and KC. The main mechanisms underlying UV-induced tumourigenesis are DNA mutation, suppression of anti-tumour immune responses, and promotion of cutaneous inflammation. However, the patterns of exposure that give rise to these tumours, and the proportion estimated to be attributable to exposure to UV radiation, differ by geographic location, skin type, and tumour type.

The association between sun exposure and melanoma is complex, and appears to differ according to the site of the tumour. A recent study supports the dual pathway hypothesis, where melanoma on sites that are less frequently exposed to the sun occurs in people with many naevi (moles), whereas melanomas on the head and neck are associated with cumulative sun exposure [33, 34]. Despite their complex association with pattern and dose of sun exposure, 75% of melanomas globally are estimated to be attributable to population exposure to excess UV radiation compared with a reference population [35]. This figure is higher in countries with higher ambient UV radiation, particularly Australia and New Zealand (96%) [35], than in those where the intensity of UV radiation is lower, such as Canada (62%) [36] and France (83%) [37]. In people with skin of colour, melanomas tend to occur on the palms of the hands, soles of the feet, and mucosal surfaces, and UV radiation is not a risk factor for these lesions [38].

With respect to KCs, SCCs have a straightforward association with cumulative exposure to UV radiation. The pattern of exposure that gives rise to BCC is less well established, but intermittent exposure in both childhood and adulthood appears to play an important role. This notion is supported by a recent meta-analysis that found stronger associations between sunburns and sunbathing in adulthood and BCC than was apparent for SCC. Sunburn in adulthood was associated with a 1.85-fold increased risk of BCC (95% CI 1.15–3.00) and a 1.41-fold increased risk of SCC (95% CI 0.91–2.18). Similar findings were reported for sunbathing in adulthood [39]. Nevertheless, one study did find that cumulative sun exposure was associated with BCC but the association with exposure before the age of 25 years of age was stronger than the association with exposure in adulthood [40]. There is little information about the link between exposure to UV radiation and risk of KC in people with skin of colour. Studies in east Asia suggest associations with measures of sun exposure, such as UV Index, outdoors occupational exposure, and lifetime exposure, but the quality of the studies is low to moderate. There are no studies in people with black skin [41].

The strong association between exposure to UV radiation and KC, combined with high prevalence of exposure, translates into a very high proportion of KCs being attributable to this exposure factor. In Canada, estimates suggested that 81% of BCCs and 83% of SCCs diagnosed in 2015 were attributable to exposure to UV radiation [39]. Easily modifiable risk factors were responsible for BCC in particular; 19% of BCCs were attributable to sunburn in adulthood and 28% to adult sunbathing (the equivalent values for SCC were 10% and 17%).

Outdoor workers are at particular risk of developing KC [42]. In a systematic review, 18 of the 19 included studies suggested an increased risk of KC among outdoors workers, although estimates were imprecise in many studies [43]. In Canada 6% of KCs in 2011 were attributed to occupational exposure to UV radiation [44]. This is similar to previous studies, where in women 1% of skin cancer (i.e. KCs and rare skin cancers) cases and 4% of skin cancer deaths were attributable to exposure to UV radiation in an occupational setting. The equivalent numbers for men were 7% of cases and 13% of deaths [45, 46].

A possible synergistic effect of simultaneous exposure to UV radiation and excessive alcohol consumption on sunburn and skin damage has previously been raised in epidemiological studies (reviewed in [47]). New work in mouse models and using human skin explants suggests that this is not due to alcohol-induced risky sun exposure behaviour, but rather that synergistic metabolic pathways induce more DNA mutations and immune dysfunction [47].

#### Skin cancers avoided by the Montreal Protocol

Estimates from the United States Environmental Protection Agency indicate that the Montreal Protocol will prevent 11 million cases of melanoma and 432 million cases of KC that would have occurred in the United States in people born between 1890 and 2100 [48]. The model estimated that cohorts born in 2040 or later will not experience any excess incidence of skin cancer caused by the effects of ozone depletion, assuming continued compliance with the Montreal Protocol. While this highlights the critical importance of the Montreal Protocol, an important limitation is that these estimates assume no changes in sun exposure behaviour and skin cancer surveillance, and no changes in population structure, such as in the distribution of skin types. Other limitations include uncertainty regarding stratospheric ozone trends, the impacts of climate change, and the action spectrum for skin cancer development.

#### Geographic variability in the incidence of melanoma

**Fig. 3:**
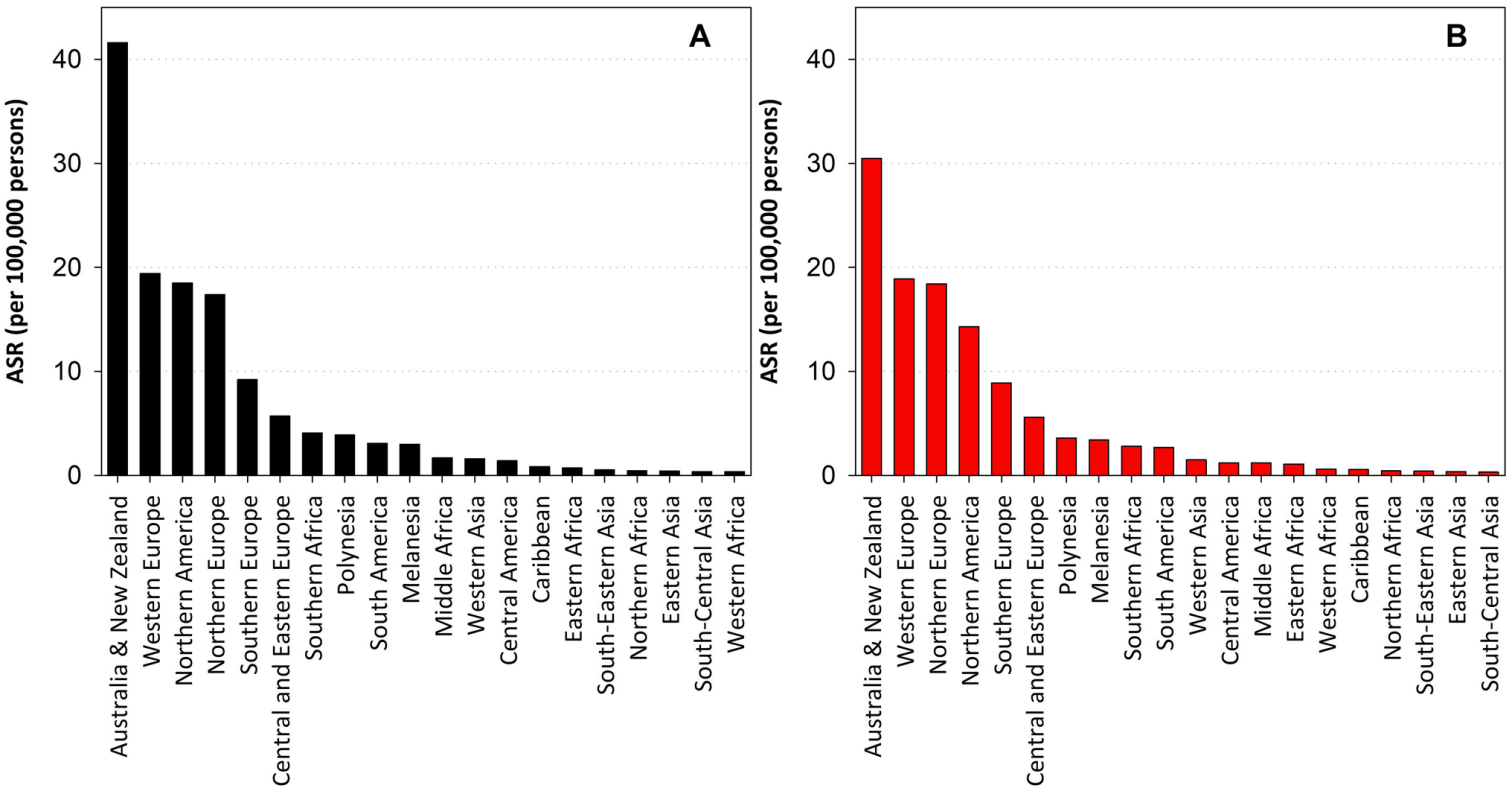
Estimated age-standardised incidence rate (world-standard population) of invasive cutaneous melanoma in the year 2020, by world region: **A** men; and **B** women (Data from the Global Cancer Observatory Database)

Worldwide in 2020 an estimated 325,000 new cases of invasive melanoma were diagnosed and 57,000 people died from melanoma [49]. The estimated age-standardised (World Standard) incidence per 100,000 people per year of invasive cutaneous melanoma was 3.8 for men and 3.0 for women. Incidence was highest in Oceania (30.1) and lowest in Africa (0.9) and Asia (0.42). Australia and New Zealand continue to report the highest incidence of all countries (Fig. 3), and the highest burden in terms of disability-adjusted life years (DALYs) lost, followed by North America and Europe [50, 51].

In 2018, it was estimated that melanoma accounted for 1.6% of all new cancer cases and was responsible for 0.6% of all cancer deaths worldwide [52]. In comparison, the most common cancer at that time (lung), excluding keratinocyte cancer, was responsible for 11.6% of cases and 18.4% of deaths. The cumulative risk of developing melanoma (birth to age 74 years, globally) was estimated to be 0.39% in men and 0.31% in women (noting that this is an average of the markedly different risks in people with light and dark skin); estimates of the cumulative risk of death from melanoma were 0.08% for men and 0.05% for women [52]. Melanoma constituted 11% of all cancer cases in Australia in 2019, and was responsible for 2.7% of deaths from cancer [53]. In Europe in 2018, melanoma accounted for 3.7% of all cancer cases (men: 3.5%; women: 3.9%), and was responsible for 2.5% of deaths from cancer (men: 3.2%; women: 1.9%) [54].

By 2040, the number of new melanoma cases globally is predicted to increase to 510,000 per year and deaths to 96,000, assuming changes in population size and age structure but no change in the incidence rates [49].

#### Trends in the incidence of melanoma, based on published reports

Trends in melanoma incidence need to be interpreted in light of changing surveillance practices. In the United States [55, 56], Australia [57], and Europe [58, 59] there has been a much greater increase in the incidence of in situ (confined to the epidermis) and thin melanomas compared with thick melanomas. The increase in melanoma incidence has also greatly outstripped increases in the mortality rate. These patterns are thought to reflect the detection of lesions that are unlikely to cause significant morbidity or mortality within a person’s lifetime, a phenomenon known as over-diagnosis, which is occurring due to the combined effect of an increase in skin examinations, lower clinical thresholds for taking a biopsy of pigmented lesions, and lower pathological thresholds for diagnosing melanomas [60, 61]. Over-diagnosis of melanomas could lead to an under-estimate of the impact of the Montreal Protocol.

Recent trends in incidence of melanoma vary across populations. Incidence increased in the United Kingdom, Norway, Sweden and Canada (1982–2015) [62], particularly the Eastern Newfoundland and Labrador provinces (2007–2015) [63], and in France (1990–2018) [64]. Of recent reports from Eastern Europe, those from Lithuania (1991–2015) [65], Ukraine (2002–2013) [66], and the Czech Republic (1977–2018) [67] described increases for all age groups and in both men and women, while a study from Hungary found increases between 2011 and 2015 followed by a significant decrease between 2015 and 2019 [68]. For Australia, New Zealand and Denmark (1982–2015) there is a recent trend of stabilising or even declining incidence, likely due to concerted efforts in primary prevention over the past 2–4 decades [62].

While incidence is very low in China and South Korea, small increases in incidence were noted (from 0.4/100,000 in 1990 to 0.9/100,000 in 2019) in China [69] and in South Korea (from 2.6/100,000 in 2004 to 3.0/100,000 in 2017) [70]. In China in 2017, the highest incidence rates were recorded for the eastern and northeast provinces compared with the western provinces, a trend which may be due to heightened awareness and greater access to medical services in these regions [71]. A study from Singapore reported very low incidence among Chinese, Malay and Indian Singaporeans [72].

A study of trends in melanoma incidence using data from the Surveillance, Epidemiology, and End Results (SEER) programme in the United States showed that across all ethnicities incidence stabilised between 2010 and 2018 (average annual percent change [AAPC], 0.39%; 95% CI − 0.40 to 1.18%), following five decades of continuous increases [73]. However, the incidence of the thickest melanomas (T4, > 4.0 mm) continued to rise (AAPC 3.32%; 95% CI 2.06–4.60%). Populations with lower socioeconomic status or from minority groups were more likely to have thicker melanomas over the time period examined, likely due to poorer access to screening and early detection activities. While the incidence of melanoma in children is very low, between 2000 and 2015 in the United States declines in incidence were reported for children aged 10–19 years, while incidence in younger children remained stable [74].

Several studies have reported different trends according to age. Studies from Canada [75], Italy [76], and England [77] report increases in incidence in older age groups, possibly at least partly due to longer life-span, but a stabilisation or decline in younger age groups. In contrast, a Finnish study of melanoma incidence in children and adolescents reported a fourfold increase between 1990 and 2014, most notable among adolescents [78]. It is unclear whether this represents a true increase or is due to changes in diagnostic criteria and/or cancer registry coverage.

#### Trends in incidence of melanoma according to age: analysis of Global Cancer Observatory data

**Fig. 4:**
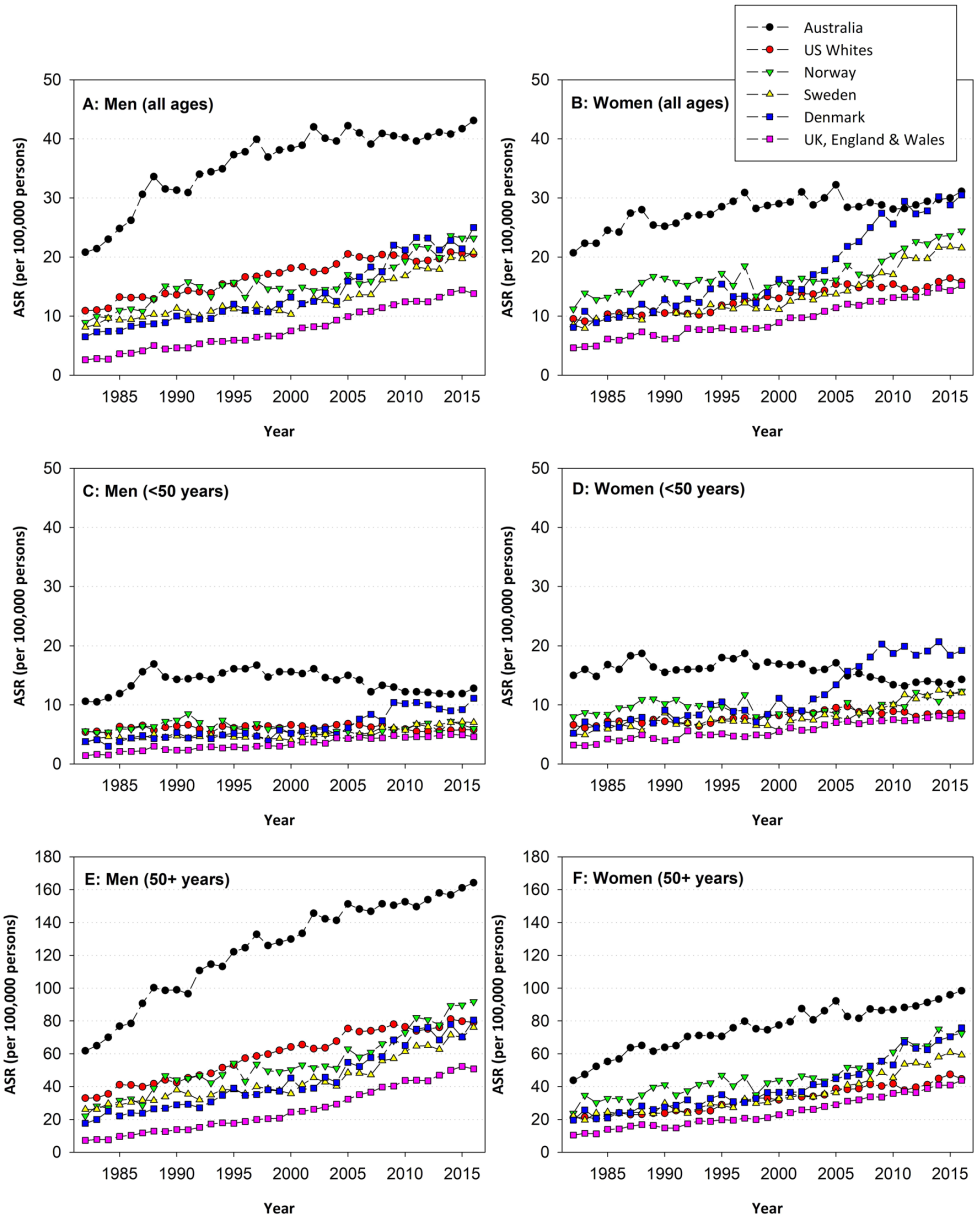
Age-standardised incidence rate (ASIR, World) of invasive cutaneous melanoma 1982–2016 in 6 populations [Australia, United States Whites, Norway, Sweden, Denmark and United Kingdom (England and Wales)] from 1982 to 2016. Trends presented separately for men and women, and for all ages and separately for those < 50 years and ≥ 50 years

It is difficult to compare trends in incidence of melanoma based on reports from the published literature due to the use of different populations for standardising age, as has been noted in the Panel’s annual assessments 2019–2021 [79–81]. We, therefore, extracted population-based registry statistics for six high-risk populations with data available for the period 1982–2016 (namely Australia, United States Whites, Norway, Sweden, Denmark and the United Kingdom) from the Global Cancer Observatory (age standardised to the World Standard Population) [82]. While incidence began to stabilise in Australia after 2005, it continues to increase in the other countries for both men (Fig. 4A) and women (Fig. 4B). However, there is marked variation with age, with modest increases among people aged less than 50 years (Fig. 4C, D) and much more notable increases among older age groups (50 years and over) (Fig. 4E, F). For Australia only, there has been a decline in incidence among younger age groups that began around 2007. In the most recent 10-year period, the estimated average annual percent change in incidence was highest for Norway (4.0% for men and 4.2% for women) and Sweden (3.8% for men and 4.0% for women). These trends are attributable to population-specific changes in time outdoors and implementation of sun-protection programmes; these will influence trends into the future as younger cohorts, who have been exposed to these behavioural changes from a younger age, enter middle and older age.

#### Trends in melanoma mortality

Trends in mortality are underpinned by changes in incidence and case-fatality rates. The latter has been decreasing markedly in some countries in recent years due to the introduction of new and highly effective systemic therapies for advanced melanoma [83], and this will continue to affect mortality rates with increasing use for earlier stage disease.

A study using data from the WHO Mortality Database covering 31 countries over the time period 1985–2015 reported an overall increase in melanoma mortality for men in all countries, in contrast with stable or declining rates in women [84]. For the most recent time period (2013–2015) the median mortality rate was 2.6 deaths per 100,000 for males and 1.6 per 100,000 for females; the highest mortality rates were recorded for Australia and Norway for men, and Norway and Slovenia for women (noting that New Zealand, which has the highest mortality globally, was not included in the report). The increase in most countries reflected increasing mortality rates in people aged 50 years or older; mortality rates were generally stable or declining in younger age groups. The latter trend likely reflects lower incidence among younger birth cohorts exposed to lower cumulative exposure to damaging UV radiation. A separate report for Spain over the period 1982–2016 showed a similar trend, with mortality rates stabilising in men and women younger than 64 years from the mid-90 s, while rates continued to rise in older age groups [85].

Recent declines in melanoma mortality have been reported for New Zealand (2015–2018) [86] and China (1990–2019) [69], but increases were reported for the Netherlands (1950–2018) [87] and Brazil (1996–2016) [88], while mortality was stable in France (1990–2018) [64] and South Korea (2014–2017) [70]. These disparate trends are difficult to interpret given heterogeneity in the introduction (and timing thereof) of new systemic treatments (particularly immunotherapy about 10 years ago) across jurisdictions.

#### Trends in the incidence of Merkel cell carcinoma

Merkel cell carcinoma (MCC) is a rare skin cancer that may be associated with exposure to UV radiation. An increase in the incidence of MCC between 1997 and 2016 has been reported for the United States, Norway, Scotland, New Zealand, and Queensland, Australia at a rate of 2–4% per year [89]. Increases have been greater in Brazil, with average annual percent change from 2000 to 2017 of 9.4% for men and 3.1% for women [90]. These findings are consistent with an earlier report covering 20 countries for the period 1990–2007 [91]. The increase in the United States has been attributed to three factors: increased detection, an ageing population, and higher exposure to UV radiation in more recent birth cohorts [92].

The cause of MCC is not well understood; the Merkel cell polyomavirus (MCPyV) is clonally integrated in up to 80% of tumours [93]. While several studies have reported more mutations in MCPyV-negative tumours (dominated by UV signature mutations) [93, 94], a new study based on 9 tumours reported more mutations in MCPyV-positive compared to MCPyV-negative tumours [95]. Because MCC is such a rare tumour, all existing studies are based on limited tumour series, and further studies using larger sample sizes are needed to understand the role of exposure to UV radiation in the aetiology of these cancers.

Survival from MCC is much lower than for melanoma (50% at 5-years for local and < 14% for metastatic disease [96]), although immunotherapy trials are reporting improved outcomes [97–99]; the costs of treatment are likely to increase if these therapies are widely adopted.

#### Trends in incidence of keratinocyte cancer

Accurately reporting the burden, incidence, and trends in KC remains a challenge. KCs are not routinely reported in most cancer registries. Further, people frequently experience more than one lesion, but this multiplicity is often not considered, with only the first lesion in a person being reported. Accounting for multiple KCs per person results in an approximately 50% increase in incidence rates [100, 101].

An analysis of Global Burden of Disease data found that in 2019 KC was the most common cancer globally, affecting almost 3 times as many people as the next most common cancer (lung—2.2 million people) [102, 103]; there were ~ 6.4 million new patients with KC. Death due to BCC is very rare, but ~ 56,000 people died due to SCC. The burden of disease, as measured by DALYs, increased by almost 25% between 2010 and 2019.

Age-standardised incidence rates of KC are highest and increasing in Australia and New Zealand [104–107], with age-standardised rates as high as 1907/100,000 (standardised to the 2001 Australian population). In Europe, increasing incidence of KC has been reported. For example, in Iceland there was a two–fourfold increase in the incidence of BCC [108] and a 16-fold increase in incidence of SCC between 1981 and 2017 [109], attributed to increased holidays to destinations with high ambient UV radiation and use of sunbeds. In Serbia between 1999 and 2015, there was an annual increase in KCs of 2.3% [110]. In the United Kingdom, SCC incidence increased by 31% and BCC by 21% between 2004 and 2014 [111]. In the United States, the incidence of KC increased from 1990 to 2004, but then remained fairly stable from 2005 to 2019 [112].

Among populations with predominantly light skin, the lifetime risk of KC is much higher in areas with high ambient UV radiation. In the United Kingdom, where ambient UV radiation is comparatively low, lifetime risk is estimated to be 20% [113]. In contrast, lifetime risk in Australia, where the ambient UV radiation is high, is estimated at 69% (73% for men and 65% for women) [114].

Benign and premalignant keratinocyte lesions caused by sun exposure add an additional burden to the already high cost of skin cancer for healthcare systems and individuals. The prevalence of actinic keratosis (benign lesions) is high and estimated to be between 25% (in a general practice population in Switzerland) and 29% (in patients attending dermatology outpatient clinics in Spain) in European populations [115, 116]. The incidence of in situ skin cancers (premalignant lesions) is also increasing, and in some countries the incidence of these lesions is increasing more rapidly than that of invasive cancers. For example, in the Netherlands the incidence of SCC increased by 6–8% per year between 2002 and 2017, compared with a 12–14% annual increase since 2010 for SCC in situ [101, 117].

#### Risks of skin cancer in people who are immunosuppressed

Immunosuppression is a risk factor for melanoma, BCC and SCC. Populations with compromised immunity at increased risk include organ transplant recipients [118], those diagnosed with HIV/AIDS (fourfold increased risk of melanoma) [119, 120], and those treated for rheumatoid arthritis (~ 1.3-fold increased risk of KC and melanoma) [121], inflammatory bowel disease (~ 1.5-fold increased risk of KC), and some lymphoproliferative disorders including non-Hodgkin lymphoma and chronic lymphocytic leukaemia (~ twofold increased risk of melanoma) [122]. In solid-organ transplant recipients the magnitude of the increased risk differs between skin cancer types: the increased risk in a high ambient UV radiation environment is two–threefold for melanoma, six–tenfold for BCC, and as high as 100-fold for SCC [123].

#### Costs associated with skin cancer management

The average paid and unpaid productivity loss per premature death from melanoma in Europe is estimated to be €450,694 [124], the second highest loss of all cancer types after Hodgkin’s lymphoma, likely due to the relatively earlier age of onset (and, thus, greater paid productivity losses).

The introduction of new systemic treatments for advanced melanoma and their increasing use as an adjuvant treatment for non-metastatic disease is causing a rise in the overall cost per capita associated with melanoma treatment globally. In the United States between 1997 and 2015, total expenditure for treatment of melanoma increased at a faster rate than for other cancers [125]. In the Netherlands malignant skin tumours were the 4th most costly cancer in 2017; drug costs increased from €0.7 million to €121 million from 2007 to 2017 [126]. The largest cost drivers in France, Germany and the United Kingdom are medications and hospitalisation and/or emergency department treatment [127]. Adverse events from the use of new treatments are also responsible for a sizable cost burden [128, 129].

A modelling study on the cost of melanoma in Europe estimated national costs ranging between €1.1 million in Iceland and €543.8 million in Germany (€2.7 billion for all European Union states) [130]. A recent study estimated the national costs of treating newly diagnosed melanoma in Australia and New Zealand for the year 2021, and reported total costs of AUD 481.6 million (€310 million^2^), and NZD 74.5 million (€43 million), respectively [131]. In Australia, the mean cost per patient was AUD 14,268 (€9,198), ranging from AUD 644 (€415) for in situ melanoma to AUD 100,725 (€64,930) for stage III/IV (advanced) disease. These costs will increase as expensive immunotherapy becomes a therapy of choice for earlier stage melanoma, either alone or in combination with targeted therapies [132].

Examining the skin to identify melanoma can lead to the detection of benign lesions, often resulting in additional treatments that may or may not be needed. A study in the United States reported on the costs of diagnosis and treatment of actinic keratoses and other benign lesions associated with screening for melanoma via total body skin examination [133]. In an analysis of 36,647 total body skin examinations in 20,270 adults, the estimated cost of treatment (including consultation, biopsy and pathology charges) for each melanoma detected was USD 32,594 (€33,614), with an additional cost of USD 7840 (€8085) to treat actinic keratoses and other benign lesions.

Given the very high and escalating costs of treatment, public health agencies have strengthened their focus on primary prevention, for which there is evidence of cost-effectiveness. A modelling study to evaluate the cost-effectiveness of prevention compared with early detection for melanoma control [134] used data from two randomised controlled trials (RCTs) conducted in Australia [135, 136]. Compared with annual clinical skin examinations (early detection), and no intervention, advice to use sunscreen daily (prevention) resulted in lower numbers of melanoma and KC cases, and significantly lower costs associated with diagnosis and treatment [134]. However, these findings may not be applicable to locations with lower ambient UV radiation, and potential costs of over-diagnosis have not yet been considered.

In Canada, the costs of KC and other rare skin cancers due to occupational exposure to UV radiation were estimated to be CAD (2011) 29 million (€22 million) in direct and indirect costs, and CAD (2011) 6 million (€4.5 million) in intangible (due to effects on quality of life) costs in 2011 [137]. These costs can be mitigated; estimates suggest that for every dollar invested in personal protective equipment and shade structures, CAD 0.49 and CAD 0.35 will be returned, respectively [138]. Another modelling study of cost-effectiveness showed that primary prevention by systematic use of sunscreen at a population level will prevent substantial numbers of new skin tumours (26% less excised KC), and save healthcare costs [134]. Among people at high risk of KC, costs for treatment of KC and actinic keratoses were reduced 1 year after treatment with topical 5-fluorouracil, showing that chemoprevention may be an option to reduce the incidence of skin cancer in this subgroup [139].

The very high and increasing costs of managing skin cancer underscore the need to protect the stratospheric ozone layer; in the absence of control of ozone-depleting substances, the intensity of UV radiation in some regions would increase to the point where many more people would be exposed to sufficient UV radiation to initiate skin cancers.

### Sunburn

Sunburn is an acute inflammatory skin reaction caused by over-exposing the skin to UV radiation, primarily the UV-B wavelengths; it is clinically manifested as erythema (redness) in people with Fitzpatrick skin types I–IV^3^ (modified from [140]), and may cause pain and blistering.

Despite the definition of sunburn varying between studies, it is a well-established risk factor for the development of cutaneous melanoma and KC [141, 142], and number of severe sunburns may be associated with increased risk of herpes zoster (i.e. shingles) [143]. Moreover, inflammation from sunburn is a health burden, independently of its association with other conditions. In the United States National Emergency Department Sample, including information about presentations to 950 hospital emergency departments from 2013 to 2015, there were 82,048 visits for sunburn, with 21% classified as severe sunburn (second or third degree burns and/or requiring inpatient admission) [144]. The average cost of an emergency department visit for sunburn was USD 1132. Presentation for all sunburns and for severe sunburns showed highest frequency in lower-income young men, and the incidence was higher in the sunnier states.

#### Trends in rates of sunburn

Data from the United States National Health Interview Surveys reveal that 34% of community-dwelling adults reported one or more sunburns in the prior 12 months in both 2005 and 2015 (sample sizes 29,250 and 31,399, respectively) [145]. The percentage of adolescents reporting sunburn was considerably higher. Between 2015 and 2017, 57% of 21,894 people aged 14–18 years reported being sunburnt at least once in the previous 12 months [146]. Sunburn was also more common in adolescents than in adults in Spain; 75% of 776 adolescents reported being sunburnt in the previous year compared with ~ 54% of 632 adults and 44% of 324 children [147]. In Germany, 22% of children aged 1–10 years surveyed in 2020 had been sunburnt in the previous year, and there was a positive association with age [148].

In some countries, there has been a reduction in the prevalence of sunburn, coinciding with increased use of sun protection behaviours. In Australia, a comprehensive skin cancer prevention campaign, SunSmart, began in 1988. Surveys conducted in the state of Victoria over the subsequent three decades, in which participants were asked about their sun protection behaviour on the weekend prior to the interview, showed a marked increase in the percentage of people using at least one sun protection behaviour (seeking shade, or using hat or sunscreen) in the first decade after SunSmart began (from 29 to 65%) and more modest increases thereafter [149]. Sunscreen use increased from 11% pre SunSmart to 68% in the 2010s. In the state of New South Wales, the percentage of people reporting often or always using sunscreen increased from ~ 30% in 2003 to ~ 40% in 2016, but there was no increase in use of hats [150]. The increase in sun protection is evident in sunburn trends. In Australian adults (*n* = 3614), the percentage reporting sunburn during the previous weekend in summer decreased from 14 to 11% between 2003/2004 and 2016/2017 [151], accompanied by an increase in the percentage of people using two more sun protection behaviours (from 41 to 45%). Sunburn occurred more frequently in Australian adolescents than in adults, but there was a decline from 20 to 15% across this period. In adults in Denmark (*n* = 33,315) a 1% annual decrease in sunburn in the previous 12 months was seen across 2007–2015, coinciding with a national sun safety campaign [152].

A birth cohort analysis of melanoma-prone families demonstrates changes in sun protection behaviour and sunburns over time. People from 17 centres in Europe, North and South America, Australia and the Middle East (*n* = 2407) were questioned about sun exposure and sunburns at various anchor points across their lives [153]. These behaviours were analysed according to birth cohort (in decades from those born in the 1910s and 1920s through to those born in the 1980s). There was a clear secular trend in the reported frequency of sunscreen use; people born more recently were more likely to use sunscreen at a younger age than those born earlier. Time outdoors on weekends at less than 20 years of age was lower in more recent birth cohorts. Within each birth cohort sunburn occurred more frequently in early *vs* later life, but more recent cohorts were less likely to experience early life sunburns.

Changes in sun exposure and prevention behaviour in some countries have been marked, which may be underpinned, at least in part, by sun protection campaigns. In the absence of the Montreal Protocol, however, it is likely that the benefits of these changes would have been less evident, as the time to sunburn would have been markedly shorter.

#### Sunburn prevalence in people of darker skin type 

Skin melanisation provides some protection against sunburn and UV radiation-induced skin cancer. Traditionally, people with dark skin (skin types V–VI) have been thought to be at very low risk of sunburn [140]. However, the difficulties of detecting sunburn erythema in people with dark skin can contribute to an over-estimation of the amount of UV radiation required to cause sunburn, and an under-estimation of sunburn prevalence [154]. Known differences in sun protection behaviours between ethnically diverse populations could also influence sunburn prevalence [155].

In a survey of people of Black African or Black Caribbean heritage living in the United Kingdom (*n* = 222 respondents), over 50% reported a lifetime history of sunburn [156], with frequencies of 47%, 54% and 71% in those self-classifying as dark, medium and light skin tone. In the United States, nearly 10% of 4157 non-Hispanic Black participants in the National Health Interview Survey 2015 reported being sunburnt in the previous year, compared with nearly 25% of Hispanic people (*n* = 5208) and 42% of non-Hispanic Whites (*n* = 19,784) [145]. These surveys suggest that sunburn occurs more frequently in people with darker skin types than traditionally appreciated, but in light of the lower severity of sunburn compared with that in people with light skin, and the extremely low risk of UV-induced skin cancer in these populations, the significance of this is unclear.

### Photodermatoses

Photodermatoses are inflammatory skin disorders that are induced or exacerbated by exposure to UV radiation and, in certain conditions, visible light [27]. Both UV-B and UV-A radiation can contribute to the development of photodermatoses. Photodermatoses fall into aetiological groups: dysregulated immune responses to UV radiation; disorders of DNA repair; intrinsic biochemical defects; photosensitivity reactions to drugs and/or exogenous chemicals; and photoaggravated disorders.

#### The burden of photodermatoses and their impact on health and psychological well-being

The lack of registry data and of consistent case definition for the most common dermatoses make it very challenging to estimate the population prevalence of photodermatoses. However, some photodermatoses, such as the immune-mediated condition, polymorphic light eruption (PLE), have been reported commonly from dermatology clinics in light-skinned populations in temperate regions, particularly during spring [157]. Comprehensive reviews of data from photodiagnostic units in dermatology departments indicate that the photodermatoses most commonly seen are PLE, photoaggravated atopic dermatitis, actinic prurigo, chronic actinic dermatitis, solar urticaria and drug-induced photosensitivity. Photodermatoses occur in dark-skin populations, although with differing frequencies and characteristics from light-skin populations [158]. In a systematic review of population-based and dermatology outpatient studies of rosacea, a photoaggravated chronic inflammatory facial condition, a global prevalence of up to 5% was estimated; however, studies in which rosacea was self-reported yielded higher prevalence than in those where the condition was determined by examination [159]. Studies of the prevalence of most photodermatoses are scarce; for example, there are no reported population-based studies in solar urticaria.

Photodermatoses involve a wide range of clinical features, which vary according to the individual condition; these include pain in the skin within a few minutes of sun exposure, severe itching, erythema, blistering, and scarring. The adverse impact on sufferers occurs both directly due to symptoms, and indirectly through restrictions imposed by sun avoidance. In a systematic review of 20 studies (2487 adult and 119 child participants), in which an assessment of quality of life or psychological well-being was performed, one-third of adults and children with photodermatoses were found to experience a very large negative impact on quality of life (Dermatology Life Quality index > 10), and anxiety and depression occurred twice as frequently as in the unaffected population [160].

#### The association between commonly used photosensitising drugs and photodermatoses and skin cancer

The pathologic mechanisms underlying drug photosensitivity are broadly classified as phototoxic or photoallergic. Oral medication-induced photosensitivity commonly involves phototoxicity, which can theoretically occur in anyone upon exposure to sufficient dose of a drug and UV radiation. Clinically, drug phototoxicity most often manifests as skin redness, swelling and burning, and can be misdiagnosed as severe sunburn.

An analysis of more than 745 million drugs dispensed in Germany and Austria between 2010 and 2017 indicated that nearly 50% had photosensitising potential, with diuretics and anti-inflammatory drugs being primarily responsible [161]. However, the global incidence of drug photosensitivity is uncertain. Analysis of the Japanese Adverse Drug Event Report database (2004–2016) found less than 0.1% of 430,587 reports concerned photosensitivity reactions [162]. A systematic review identified 1134 reported cases of suspected drug phototoxicity associated with 129 oral drugs [163]. However, the quality of the evidence for an association with drugs is low; fewer than 25% of studies performed phototesting, and only 10% confirmed the diagnosis with drug challenge–rechallenge testing. In a report of 2243 patients with photodermatosis evaluated at a photodiagnostic unit, 5% were diagnosed with photodermatosis induced by oral medication. All underwent broadband UV radiation testing and monochromatic testing to wavelengths from 300 to 600 nm (i.e. in the UV-B, UV-A, and visible spectra). UV-A was the main provoking waveband with UV-B contributing in 15% of cases [164].

It is possible that commonly prescribed photosensitising drugs may induce skin cancer. Some mechanisms by which drugs induce acute photosensitivity are also relevant for skin cancer induction, such as promotion of UV-induced DNA damage. In a nested case–control study, using data from the Danish Cancer Registry, of people with their first diagnosis of BCC (*n* = 71,533) or SCC (*n* = 8629) and population controls (*n* = 1,430,883), there was an increased risk of KC with long-term use of hydrochlorothiazide (a diuretic medication commonly used for treatment of high blood pressure); adjusted odds ratios (ORs) for high use *vs.* never use were 1.29 (95% CI 1.23–1.35) for BCC and 3.98 (95% CI 3.68–4.31) for SCC [165]. This led to the European Medicines Agency recommending that advice on increased risk of KC should be included in hydrochlorothiazide product information [166]. Further studies, based in different geographic locations and demographic groups, reveal heterogeneous and conflicting results for an increased risk of KC and melanoma with hydrochlorothiazide use [167–170] [171]. Given its potential public health significance, this issue needs to be resolved.

### Eye diseases associated with exposure to UV radiation

Exposure to UV radiation, either directly or through intermediate factors, is associated with increased risk of cataract of the lens, pterygium, squamous cell carcinoma of the cornea and/or conjunctiva, photokeratitis (affecting the cornea) and photoconjunctivitis, pinguecula, and possibly intraocular melanomas, macular degeneration and glaucoma. This section assesses evidence available since our last assessment [6] on conditions that are directly related to exposure to UV radiation.

The superficial layers of the eye are exposed to UV radiation and incur damage through the same pathways of DNA damage and production of reactive oxygen species as is seen in the skin. When the individual is in an upright position and the sun is overhead, there is some inherent protection from exposure to UV radiation provided by the protrusion of the brow, the eyebrows, and the eyelids. These provide less protection at other body positions (e.g. lying down), or when the sun is at a lower angle [172, 173]. Wearing a hat and using shade can also reduce exposure, while high surface albedo can increase exposure; large and wraparound sunglasses that block both UV-A and UV-B radiation provide good sun protection [174–176]. UV wavelengths also penetrate to the deeper structures of the eye (reviewed in a previous assessment [177]). The cornea absorbs wavelengths below 295 nm, but allows longer wavelengths to reach the iris and lens. In adults, the lens of the eye absorbs all wavelengths below 370 nm, and greater than 98% of wavelengths between 370 and 400 nm, with higher absorbance in the posterior part of the lens [178]. Over time, the chemical changes induced by that absorption—direct UV-B induced damage and (indirect) UV-A induced photo-oxidation of soluble lens proteins—cause clouding of the lens; i.e. cataract [178]. In young children, the lens may transmit a greater proportion of shorter UV wavelengths, allowing these to reach, and potentially damage, the retina.

#### Trends in the prevalence and incidence of cataract 

Cataract is the major eye condition associated with long-term exposure to UV radiation. The main types of cataracts, as defined by their location in the lens, are nuclear, cortical, or posterior subcapsular. In many cases, there is a mixed phenotype and, within any individual, the two eyes may contain cataracts with a different predominant phenotype. The two subtypes most clearly associated with exposure to UV radiation are nuclear and cortical cataracts.

According to the latest reports of the Vision Loss Expert Group of the Global Burden of Disease Study, cataract was the leading cause of blindness between 1990 and 2015 around the world, accounting for 35% (95% CI 26–44) of the total blindness in 2015 [179–183]. Projections to 2020 from several countries/regions indicate that cataract would remain the main cause of blindness in 2020 [179–184]. Compared with global figures, the proportion of moderate to severe vision impairment caused by cataract was estimated to be higher in East Asia [179], South-east Asia [181], Oceania [181] and Sub-Saharan Africa [183] where exposure to sunlight may be higher and access to suitable medical care may be limited. The disability from cataract (measured in DALYs) increased from 3.5 million in 1990 to 6.7 million in 2019—an increase of 191% [185].

New studies further demonstrate the high prevalence of cataract. In the cross-sectional Ural Eye and Medical Study set in a rural area of Russia, the prevalence of cataract was 45% in people aged ≥ 40 years (of 5899 participants, 81% of eligible residents). Nuclear and cortical cataracts affected 38% and 15% of participants, respectively [186]. A population-based study conducted in Finland found that the prevalence of cataracts increased from 8.1% (95% CI 7.8–8.5) to 11.4% (95% CI 10.9–11.9) among individuals ≥ 30 years between 2000 and 2011 [187]. The annual average incidence over the 11-year period was estimated to be 109 cases per year per 10,000 people (95% CI 104–114) [187]. The cumulative incidence over a similar time period (baseline 2004–2006; follow-up 2011–2013) was greater in Singapore; in the Malay Eye Study the age-standardised cumulative incidence of nuclear and cortical cataract over this time period was estimated to be 13.6% and 14.1% (equating to an annual average crude incidence of 227 and 189 cases per year per 10,000 individuals), respectively [188].

Greater exposure to UV radiation has been clearly linked to an increased risk of cataract (reviewed in [178]). A recent study provides additional supporting evidence. In a population-based cross-sectional study in three different rural areas of India (*n* = 12,021), 33% of participants aged 40 years and older had a cataract in at least one eye [189]. Compared with the lowest quintile of a lifetime effective sun exposure score (calculated taking into account the years of exposure, hours of sun exposure accounting for type of headgear used (none, caps, hats, umbrellas, veils, sunglasses)), the prevalence of cataract was significantly higher in the 3rd, 4th and 5th quintiles of exposure. Those in the fifth quintile were 9 times more likely to have cataracts than those in the first quintile (adjusted OR 9.4; 95% CI 7.9–11.2), rising to nearly 26 times more likely in analyses confined to the highest altitude region (Guwahati/Hills region). Differences in exposure to UV radiation, solar angle and sun protection behaviours each had an additional influence on prevalence of cataracts. Nevertheless, in data from the 2008–12 Korea National Health and Nutritional Examination Survey of economically active people, there was no significant association between higher sunlight exposure (≥ 5 h vs*.* < 5 h/day in the sun without sunglasses or hat) and medically diagnosed cataract (adjusted OR 0.88, 95% CI 0.77–1.00) [190].

Globally, and across diverse individual regions for which there are recent data, the incidence of cataract continues to increase, at least partly due to ageing populations. Where there is good access to high-quality medical care, including cataract surgery, this may not contribute greatly to the burden of disability. However, in many regions, cataract remains a leading cause of blindness, resulting in considerable morbidity due to vision loss and its sequelae (e.g. falls) [191].

A recent study has estimated the effectiveness of the Montreal Protocol in preventing eye diseases, with a focus on cataract. It was estimated that the implementation of the Montreal Protocol with all of its Amendments and adjustments compared to a scenario of no control of ozone-depleting substances, will prevent 63 million cataract cases in people born in the United States between 1890 and 2100. When the comparison scenario is the original Montreal Protocol, this figure is 33 million fewer cases of cataract, demonstrating the importance of the ongoing strengthening of the Protocol [48].

#### Prevalence of pterygium

Pterygium is a non-cancerous, self-limiting pink, fleshy tissue growth on the conjunctiva, that is initially induced by exposure to both UV-B and UV-A radiation. The mechanisms of how and why pterygium is self-limiting have been clarified by recent studies [192]. As this condition commonly occurs in surfers who are exposed to significant amounts of sunlight, it is often referred to as ‘surfer’s eye’. The impact of pterygium on vision is minimal unless it reaches the cornea, but it is painful to remove and often recurs after surgical removal.

Evidence suggests that the prevalence of pterygium has slightly increased in recent years. In a recent meta-analysis of 55 studies (including data from > 400,000 people in 24 countries), the overall prevalence of pterygium was estimated at 12% (95% CI 11–14) [193]. However, studies included a diverse range of age groups and not all were population based. The reported prevalence was higher than that from a 2013 meta-analysis based on 20 articles from 12 countries (10.2%; 95% CI 6.3–16.1%) [194].

Studies from Brazil demonstrate the high variability in prevalence estimates according to location and study methods. In a population-based study in the Brazilian Amazon, including 2041 people (86% of those eligible to participate) aged 45 years and over, the prevalence was 58% [195]. The recent meta-analysis estimated prevalence in Brazil to be 52.0% (in an ophthalmic clinic-based study in Manaus, age range 21–61 years); 21.2% in the Amazon rainforest (population-based study of people 11 years and older); 18.4% in the Brazilian rainforest (population-based, no age data); and 8.1% in São Paulo (population-based, median age 49.6 years) [193]. Such variability challenges the simple combining of estimates across studies. However, the prevalence of pterygium seems to be modest and consistent across various regions of China, estimated to be approximately 6% [196, 197]. A population-based cohort study, the Gutenberg Health Study, including the German city of Mainz and the surrounding regions (latitude 50°N), found a very low prevalence of pterygium with an estimate for the weighted prevalence of 0.9% (95% CI 0.8–1.2) in people aged 40–80 years [198]. The presence of pterygium was associated with male sex, higher age, and migration from Arabic-Islam countries, the former Soviet Union, and former Yugoslavia. In a slightly higher latitude region, including both an urban and rural multi-ethnic population in Ufa city and surrounds in Russia, the prevalence of pterygium was 2.3% (95% CI 2.0–2.7) among people over 40 years [199]. Risk factors for pterygium were rural residence, higher age, and lower level of education.

The incidence of pterygium has been reported in two longitudinal studies. In Southern India, which lies within the ‘pterygium belt’ (37° north and south of the equator where pterygia are most common [200]), the age- and sex-adjusted incidence was 25.4 per 100 person years (95% CI 24.8, 25.7) over a 15-year period in residents (*n* = 2290) of rural areas aged 30 years and older at baseline [201]. The overall incidence rate in 6122 adults aged 40 years and over was considerably lower (age-adjusted 6-year incidence = 1.2%; 95% CI 1.0–1.6%) over 6 years of follow-up in the Singapore Epidemiology of Eye Diseases Study [202].

In a meta-analysis of risk factors for pterygium several factors associated with solar exposure of the eyes increased the risk of pterygium, including spending more *vs.* less than 5 h outdoors per day (OR 1.24; 95% CI 1.11–1.36), or having outdoor vs*.* indoor occupations (OR 1.46; 95% CI 1.36, 1.55). Furthermore, in a recent study reporting on findings from the Korean National Health and Nutritional Examination Survey, an average of ≥ 5 h/day in the sun without sunglasses or hat, compared to < 5 h, was associated with an increased risk of pterygium in women (OR 1.47, 95% CI 1.16–1.73) but not men (OR 0.88, 95% CI 0.70, 1.10) [190]. There was a dose response apparent, with greater time outdoors associated with higher risk. Importantly, in the meta-analysis, wearing sunglasses reduced the odds of pterygium by approximately 50% (OR 0.47; 95% CI 0.19–0.74) [193]. In support of this finding, in a longitudinal study of young adults in Australia, wearing sunglasses for at least half of the time outdoors resulted in a significantly greater decline in the area of conjunctival UV fluorescence, a biomarker of sun exposure, over 8 years compared to never or seldom use [203].

#### The link between exposure to UV radiation and intraocular melanoma 

Intraocular melanoma is the most common type of cancer that develops within the eyeball, but it is rare compared to cutaneous melanoma. Intraocular melanomas predominantly occur on the uvea and conjunctiva, but uveal are considerably more common than conjunctival melanomas.

Exposure to sunlight, light pigmentation of the eye and skin, and living at high latitudes are often reported as risk factors for both types of intraocular melanoma, akin to melanoma of the skin. We have previously assessed the epidemiological and genetic evidence regarding the role of UV radiation in the aetiology of intraocular melanoma, with more convincing evidence for conjunctival vs*.* uveal melanoma [177]. Recent genetic studies provide further evidence of similarity of intraocular to cutaneous melanoma and, thus, a possible causal role of exposure to UV radiation. A study comparing the genetic changes in uveal melanomas with those in cutaneous melanomas has shown many shared mutations, including UV signature mutations, suggesting that some uveal melanomas may be UV dependent [204]. In a similar study, tissue samples from conjunctival melanomas displayed evidence of genetic changes consistent with UV-related damage similar to those found in melanoma of the skin [205].

Published recent incidence data for intraocular melanoma are available from four developed countries that have well-established cancer registries: United States, Canada, Australia and Ireland (Table 1). The age-standardised incidence rate ranged from 3.3 per million in Canada [206] to 9.5 per million in Ireland [207], but rates are not directly comparable due to the use of different populations for age standardisation and different periods of observation. On average the age-adjusted incidence increased by 0.5% per year in the United States between 1973 and 2013 (*p* < 0.05) [208]. In Canada, there was minimal change from 1992 to 2010 [206]. In Australia, there was an increase of 2.5% per year from 1982 to 1993, followed by a decrease of 1.2% per year from 1993 to 2014 [209]. Thus, in these countries, the incidence of intraocular melanomas has remained relatively constant over time, in contrast to that of cutaneous melanomas.

**Table 1:** Age-standardised incidence rates of intraocular melanomas across developed countries and regions

Study	Country/Region	Period	Age-standardised incidence rate per million person years (95% CI)
Uveal melanoma			
Aronow et al. [208]	United States	1973–2013	5.2 (5.0, 5.4)^a^
Baily et al. [207]	Ireland	2010–2015	9.5 (8.4, 10.7)^b^
Ghazawi et al. [206]	Canada	1992–2010	3.3 (3.2, 3.5)^c^
Beasley et al. [209]	Australia	1982–2014	7.6 (7.3, 7.9)^d^
Conjunctival melanoma			
Ghawazi et al. [210]	Canada	1992–2010	0.32 (0.28, 0.37)^c^
Virgili et al. [211]	Europe	1995–2007	overall 0.42^e^
Virgili et al. [211]	Northern Europe	1995–2007	0.81 (0.59, 1.09)^e^
Virgili et al. [211]	UK and Ireland	1995–2007	0.40 (0.36, 0.45)^e^
Virgili et al. [211]	Central Europe	1995–2007	0.59 (0.51, 0.68)^e^
Virgili et al. [211]	Southern Europe	1995–2007	0.35 (0.26, 0.47)^e^
Virgili et al. [211]	Eastern Europe	1995–2007	0.27 (0.22, 0.33)^e^

^a^Age-adjusted to the US population 2000

^b^Age-standardised using the 1976 European standard population

^c^Age-standardised using the World Standard Population

^d^Age-standardised using the 2001 Australian standard population

^e^Age-standardised using the European standard population

The incidence of conjunctival melanoma was substantially lower compared to uveal melanoma in incidence studies from Canada and Europe, replicating previous findings. The age-standardised incidence rate of conjunctival melanoma was 0.32 cases per million people per year (age standardised to the World Standard Population) between 1992 and 2010 in Canada [210], while it was 0.42 cases per million people per year (age standardised to the European Standard Population) in Europe [211].

#### Damage to the eye from drug-induced phototoxicity

A number of drugs absorb in the UV range and have phototoxic side effects affecting various structures in the eye [212]. For example, fluoroquinolone antibiotics such as ciprofloxacin and norfloxacin (used to treat ocular infections), in the presence of UV-A radiation, caused damage to epithelial cells (in cell culture) and proteins of the lens. Exposure of the eye to UV-A radiation while using these compounds could accelerate the development of cataract [213]. Use of ophthalmic formulations containing ketoconazole, diclofenac, or sulphacetamide were found to be toxic or irritating in the presence of UV-A radiation [214]. While there is growing awareness of cutaneous photosensitivity in relation to systemic drugs, the focus for eyes appears to have been on exposure to UV-A radiation in conjunction with topical medications. It will be important to better understand the potential photosensitisation resulting from both topical and systemic drugs for the eye, given its vulnerability to damage from exposure to UV radiation and the clear protection that sunglasses provide.

### Non-skin cancer-related harms of UV-induced immune suppression

#### Increased risk of systemic infections and reduced vaccine effectiveness 

Hart and Norval [12] hypothesised that vaccination through acutely or chronically sun-exposed skin (e.g. the upper arm, a common site for intramuscular vaccination) may result in a less effective immune response compared to unexposed skin (e.g. buttock). However, there remains little confirmatory evidence for this at present. In a cluster randomised trial in children in rural South Africa, an intervention to protect vaccinees from solar UV radiation did not result in higher antibody levels following a measles booster [215]. However, in a small clinical trial testing the immune response to a novel antigen (keyhole limpet haemocyanin)—although higher natural exposure to UV radiation was not associated with a change in antigen-specific antibodies—there was a reduced T-cell response [216]. Any effect of exposure to UV radiation may be more important for vaccines that rely on a cell-mediated, rather than humoral (antibody), response to vaccination; e.g. Bacille Calmette Guerin (BCG) for tuberculosis, particularly in low latitude (higher UV radiation) locations.

#### UV radiation and reactivation of viruses

The association of intense exposure to UV radiation with subsequent reactivation of Herpes simplex virus 1 (HSV), causing cold sores of the lip, is well described (reviewed in [177]). The presence of IgM class antibodies to HSV reflects recent viral activity, either primary or recurrent infection [217]. In a recent study from Sweden, the odds for anti-HSV IgM positivity were nearly twofold higher (odds ratio = 1.99 per mean MED difference) in summer than in winter (mean MED difference was 9.967 equivalent to 2093.1 J m^−2^), consistent with UV-induced reactivation of HSV, with or without the manifestation of cold sores [217].

There is considerable current interest in another herpes virus, Epstein–Barr virus (EBV), in relation to risk of multiple sclerosis (MS), nasopharyngeal carcinoma and other diseases. Results from a recent study from Hong Kong [218] suggest that higher personal sun exposure is associated with reactivation of EBV. The measures of personal exposure included ambient UV radiation at the date of blood collection, serum 25(OH)D concentration, and self-reported duration of sunlight exposure (hours/day) over four life periods (6–12 years, 13–18 years, 19–30 years, and 10 years prior to recruitment). EBV reactivation was measured as seropositivity to EBV viral capsid antigen (VCA) IgA. Only duration of sunlight exposure at 19–30 years and 10 years prior to recruitment (for ≥ 8 h compared to < 2 h, OR 2.44, 95% CI 1.04–5.73, OR 3.59, 95% CI 1.46–8.77, respectively) were associated with increased odds of VCA-IgA seropositivity (inferred as evidence of reactivation). Reactivation of EBV may be a trigger of relapses in MS [219]. Thus, higher levels of sun exposure, leading to EBV reactivation, might be expected to also be associated with relapse. However, previous research suggests that higher sun exposure (over the life-course prior to MS onset) is associated with fewer relapses in people with MS [220]. Nevertheless, the time course of sun exposure may be of importance; higher sun exposure earlier in life may be protective for the development of MS through immune mechanisms, but after EBV infection higher sun exposure may be associated with increased risk of relapse through reactivation of EBV. Datasets are available that could test this hypothesis.

Varicella zoster virus is a herpesvirus that causes chicken pox during primary infection and shingles on reactivation. Using data from Thailand, a recent study examined seasonal variation in case reports of chickenpox and shingles [221]. Both chickenpox and shingles showed strong seasonality. Chickenpox was characterised by outbreaks beginning during November and December, with seasonal peaks in February and March (with deep troughs from June to October). The amplitude of the seasonal effect decreased closer to the equator. Shingles showed a peak in May–June, with a shallow trough in February–March and a deep trough in October–December. Again, higher latitudes had more pronounced seasonal cycles. Changes in ambient UV radiation were the main driver of the seasonal cycle for shingles reactivation, but not chickenpox, consistent with an effect of UV radiation on reactivation but not primary infection with Varicella zoster virus.

## Benefits of exposure to UV radiation

Sun exposure has numerous benefits, many of which are mediated by exposure to UV radiation, and some by exposure to other wavelengths. People need to be able to safely spend time outdoors to gain these benefits. The Montreal Protocol has likely enabled the benefits to be gained, by preventing the intensity of ambient UV radiation from increasing to an extent where it would have been very difficult for light-skinned people in particular to spend time outdoors without markedly increasing their risk of UV-induced skin and eye diseases.

### Health benefits of greater time outdoors and sun exposure

We have previously reported on the evidence of health benefits of exposure to sunlight for autoimmune and cardiovascular diseases, as well as myopia and some cancers [6]. It is challenging to generate high-quality evidence from human studies of benefits of exposure to UV radiation, primarily because it is difficult to capture accurate exposure data over a relevant time period. In addition, it is challenging to determine which wavelengths of sunlight are most important and further, how much of any effect of exposure to the sun is through vitamin D vs*.* non-vitamin D pathways. Determining whether associations are causal, and thus whether the balance of risks and benefits of sun exposure needs to be reconsidered, will require accumulation of evidence across epidemiological and mechanistic studies [222]. Recent studies have been largely cross-sectional, and/or used population-level exposures such as sunshine duration [223], ambient UV radiation or location [224], or remote sensing of green space coverage [225]. From these studies, benefits of higher green space coverage, longer duration of sunshine, or higher individual levels of sun exposure have included lower blood pressure in adults [226] and children [225], and reduced prevalence of obesity [223] and depression [227].

In a large study (*n* = 342,457) of patients undergoing dialysis in 2189 facilities across the United States, monthly average ambient UV irradiation at the clinic location had a linear inverse association with monthly average pre-dialysis systolic blood pressure, including after adjustment for ambient temperature [224]. The effect size was greater in Whites than in Blacks. These data are consistent with new analyses of the Melanoma in Southern Sweden study in which women with low or moderate past sun exposure (assessed by questionnaire including items on deliberate sun bathing, use of a sun bed, and travel for sunny holidays) had a greater risk of being prescribed anti-hypertensive medication by their physician than those with higher sun exposure; the association persisted after adjustment for being a smoker, exercise category, BMI, and education (adjusted OR 1.41, 95% CI 1.3–1.6; adjusted OR 1.15, 95% CI 1.1–1.2, respectively, for low and moderate sun exposure) [228]. In a recent study from South Korea, there were fewer cardiovascular (adjusted hazard ratio (HR) 0.68, 95% CI 0.49–0.94) and cerebrovascular (adjusted HR 0.60, 95% CI 0.47–0.77) events over 11 years in patients with vitiligo who had received long-term narrowband UV-B phototherapy (≥ 100 sessions) compared with those who had received < 3 phototherapy sessions [229]. There is accumulating evidence, including from small clinical trials, that UV-A (and possibly UV-B) irradiation influences blood pressure (and cardiovascular disease risk) through release of nitric oxide from stores in skin [230–232].

There is compelling evidence from multiple studies supporting reduced risk of myopia with greater exposure to UV radiation and/or high intensity visible light. Longitudinal cohort studies from China [233], the Netherlands [234], and Australia [235] show that more time spent outdoors during childhood, measured using a variety of metrics, was associated with reduced risk of developing myopia in childhood and young adulthood. Furthermore, two studies found that greater outdoor activity in childhood could reduce the adverse effect of higher levels of screen time [233, 234]. Another study showed that lower area of conjunctival autofluorescence was associated with a greater risk of developing myopia between ages 20 and 28 years [236], suggesting that the protective effects of sun exposure may continue into young adulthood. In a cross-sectional analysis within the Singapore birth cohort study, more time outdoors, but not light levels or the timing and frequency of light exposure, was associated with lower odds of myopia [237]. Another study found that greater green space coverage was associated with lower prevalence of myopia [238]. While more time outdoors seems well-established as protective for the development of myopia, details of optimal exposure to minimise myopia are not fully elucidated. Of note, the effect of greater exposure to UV radiation appears to be distinct from any effect of varying focal length during time outdoors [239].

There is now considerable evidence that there may be benefits of spending more time outdoors/sun exposure for the onset and progression of MS in addition to those ascribed to vitamin D (see below). A recent multi-ethnic case–control study confirmed a protective effect of higher sun exposure on risk of developing MS in white populations, and extended this to show the benefits were also apparent for blacks and Hispanics [240]. In contrast, benefits of higher 25(OH)D concentration were apparent only in United States Whites, possibly because 25(OH)D concentration is a better indicator of recent sun exposure in people with lighter skin. A case–control study in Canada, Italy, and Norway demonstrated that an accumulation model for sun exposure to age 15 years, rather than a critical periods model, provided the best fit for the protective effects of higher sun exposure on risk of MS in adulthood [241]. Importantly, among those who spent a lot of time outdoors in summer, use of sun protection did not alter MS risk. These findings highlight the need to provide balanced sun exposure messages that take account of geographical differences in weather patterns, skin pigmentation, and cultural practices [241]. Another case–control study showed a strong protective effect of greater time outdoors in the summer prior to diagnosis or during the first year of life, as well as higher ambient UV radiation, on the risk of developing paediatric MS [242].

The focus in relation to MS has been largely on the risk of developing the disease. There is new evidence that higher sun exposure prior to developing MS, and increasing sun exposure post-diagnosis, are associated with a more favourable post-diagnostic disease course [220, 243] [244], although there is some evidence that sun exposure may be detrimental for people with MS who have a sun-sensitive genotype [243]. A trial of narrowband UV-B radiation in people with clinically isolated syndrome to prevent the development of MS [245] found a lower risk of progression to MS in people receiving phototherapy than in the control group, although in this small study this was not statistically significant. Analyses of data from this trial have since revealed some novel potential pathways activated by narrowband (311 nm) UV-B, including transient changes in both the number of circulating leukocytes [246] and the production of pro-inflammatory cytokines [247]. Given the non-solar spectrum used, this is likely to be most relevant to a treatment setting, but it does indicate the possible importance of UV-B radiation for this condition.

There is also recent evidence that exposure to higher intensity of UV radiation during early life may protect from the development of type 1 diabetes—an autoimmune disease of the pancreas. In a data-linkage-based cohort study of 29,078 children in Western Australia (~ 6% of whom were diagnosed with type 1 diabetes by age 16), higher ambient (erythemally weighted) UV radiation was associated with reduced risk of developing type 1 diabetes, but only in males and only for UV radiation during the 3rd trimester and 1st year of life [248]. The authors concluded, assuming a causal association, that for every 100 kJ m^−2^ increase in total lifetime dose of ambient UV radiation dose, the relative risk of developing type 1 diabetes in males decreased by 29%.

Emerging evidence suggests that higher antenatal sun exposure may reduce the risk of pre-term birth [249] and learning disabilities [250]. However, higher pre-delivery ambient temperatures have been linked to increased risk of pre-term birth [251, 252], complicating analyses where data on personal exposures and potential confounders are not available, and multiple environmental exposures acting at different time-points during pregnancy need to be considered. Additional research will be required to clarify the role of personal sun exposure during pregnancy on the many facets of the health of the offspring.

Exposing the skin to UV radiation enhances feelings of well-being, possibly through the release of beta-endorphins following UV-B-induced DNA damage in keratinocytes [253]. This could provide a biological underpinning to an ‘addiction’ to tanning. In addition, serotonin is produced in the brain in response to bright sunlight [254], with this pathway potentially important for seasonal variability in mood and seasonal affective disorder. Observational studies show links between higher exposure to sunlight and reduced risk of depressive disorders, but confounding and reverse causation are possible explanations for these findings. However, artificial light therapy is established as a treatment for disorders such as seasonal affective disorder [255], and a recent experimental study confirms the benefit of sunlight. The single-blind clinical trial tested the effect of sunlight therapy (exposure of sun-protected forearms or calves to sunlight on sunny days (at least 10,000 lx) in Taiwan for an accumulated minimum of 30 min/day for a total of 14 days in 4 weeks) on depression in participants who were at least 1 month post-stroke. Testing at 1 month after the completion of the intervention showed a significant reduction in the depression score in the group receiving the sunlight therapy compared to a control (usual treatment) group [256].

There is growing interest in better understanding the potential benefits of sun exposure and the pathways and wavelengths involved. This information is critical to providing appropriate messaging to different populations on safe sun exposure to balance harms and benefits.

### Vitamin D

Perhaps the best known benefit of sun exposure to the skin, driven by UV-B radiation, is the synthesis in the skin of vitamin D. Most populations derive very little of their vitamin D needs from diet, thus relying primarily on this UV-B-induced synthesis.

#### The role of vitamin D in health outcomes

Vitamin D is best known for its role in musculoskeletal health. Vitamin D status is defined according to the blood concentration of 25(OH)D, with a concentration of < 50 nmol L^−1^ commonly considered vitamin D deficient (including here unless specifically stated otherwise). Vitamin D deficiency as defined at this concentration is associated with increased risk of hip fractures in people aged 60 years and over [257]. It has been estimated that, assuming this association is causal, approximately 8% of hip fractures occurring in adults aged ≥ 65 years in Australia are attributable to vitamin D deficiency (25(OH)D < 50 nmol L^−1^) [258]. Falls in older adults have also been linked to 25(OH)D concentration < 50 nmol L^−1^ [259]. Despite the established link between vitamin D and musculoskeletal health, the optimal 25(OH)D concentration to minimise fractures and falls is uncertain. Meta-analyses of randomised controlled trials (RCTs) show that vitamin D supplementation alone is only of benefit in people who are vitamin D deficient (< 50 nmol L^−1^) [260] or that it has no effect [261]. The Vitamin D and Omega-3 Trial in the United States did not find any benefit of supplementing older adults with vitamin D for 5 years on fractures, including in people whose baseline 25(OH)D concentration was < 50 nmol L^−1^ [262], but there was insufficient power to assess the effect in people with more severe vitamin D deficiency. These findings collectively suggest that the risk of falls and fractures may not increase until 25(OH)D concentration drops to the range currently considered to be severely deficient (< 25 nmol L^−1^).

The importance of vitamin D for other health outcomes remains unclear. Observational studies are prone to confounding or reverse causality. This can be overcome by Mendelian randomisaton (MR) studies (which examine the association between genetically determined, rather than measured 25(OH)D concentration, and health outcomes), although most MR studies have not allowed for non-linear associations between genetically predicted 25(OH)D concentration and disease, and are mostly silent on the consequences of severe vitamin D deficiency. RCTs provide additional information regarding the causality of associations. However, RCTs test the effect of a particular supplement dose and dosing regimen in a specific population for a set length of time during one life period. The absence of effect in an RCT cannot, therefore, be used as proof of lack of a causal association. With these cautions in mind, we present below a summary of recent evidence for some common disease conditions.

Low 25(OH)D concentration has been consistently linked with increased risk of depression in observational studies [263]. MR studies suggest that this association may not be causal [264, 265], and it is likely that adequate vitamin D status is a good marker of exposure to other beneficial wavelengths in sunlight that have an important effect on mood. Data from RCTs are somewhat inconsistent. A meta-analysis revealed an effect of vitamin D on negative emotion, but with very high heterogeneity, and the effect was predominantly seen in people who were vitamin D deficient or who were depressed at study baseline [266]. In support of this, a very large trial in the United States among adults without depression at baseline did not find any benefit of 5 years of vitamin D supplementation [267].

There are similarly inconsistent findings for type 2 diabetes mellitus (T2DM). A meta-analysis of observational studies found that each 1 standard deviation (SD) higher 25(OH)D concentration was associated with a 20% lower risk of T2DM (*p* < 0.001), but a genetically predicted 1 SD increase was not significantly associated with T2DM [268]. An MR study in a Chinese population also found no association between genetically predicted 25(OH)D and T2DM [269]. An RCT in which 2423 people with prediabetes were supplemented with 4000 IU of vitamin D per day for ~ 2.5 years did not find a statistically significant reduction in the incidence of T2DM, although it is important to note that the mean 25(OH)D concentration at baseline was in the sufficient range (70 nmol L^−1^) and only 22% of participants were vitamin D deficient (< 50 nmol L^−1^) [270, 271]. In a meta-analysis of RCTs in people without T2DM, vitamin D supplementation significantly reduced fasting glucose and fasting insulin but had no effect on incident T2DM overall or in progression from prediabetes to T2DM [272]. It is plausible that the findings in the observational studies reflect a non-vitamin D pathway of sun exposure, whereby higher 25(OH)D concentration is an indicator of having received sufficient sun exposure to gain the other benefits. This hypothesis is supported by mouse studies that suggest UV radiation-induced release of nitric oxide from the skin can suppress the development of glucose intolerance and hepatic lipid accumulation [273].

Observational studies consistently demonstrate inverse associations between 25(OH)D concentration and cancer incidence [274], but confounding and reverse causality are possible explanations for this finding, and this is not supported by MR studies [275] or RCTs [274]. Evidence is emerging, however, for a possible beneficial effect of vitamin D supplementation on cancer mortality [274], [276].

Case–control and cohort studies support an increased risk of MS with low 25(OH)D concentration [277], and this is supported by MR studies [278]. The association is less clear for other autoimmune diseases such as type 1 diabetes mellitus [279] and inflammatory bowel disease [278]; although there are suggestive protective effects, confidence intervals are wide and small effects cannot be ruled out. In terms of infectious diseases, observational studies [280], RCTs [281], and MR studies [282] indicate that low 25(OH)D increases risk and severity [283] of respiratory tract infection.

An analysis including over 500,000 participants found strong evidence for a non-linear association between serum 25(OH)D concentration and coronary heart disease, stroke, and all-cause mortality. This was supported by an MR analysis, which suggested the risk associated with low genetically predicted 25(OH)D was only evident in those with measured 25(OH)D below 40 nmol L^−1^ [284]. However, a recent re-analysis (published after the reference list for this paper was finalised), using different model assumptions, found no significant association with genetically predicted 25(OH)D and mortality outcomes, irrespective of 25(OH)D concentration, suggesting that the earlier analysis may have generated incorrect findings (https://www.pubmed.ncbi.nlm.nih.gov/36528346/).

Collectively, these findings suggest that vitamin D plays a causal role in some health outcomes, in addition to falls and fractures. However, there is no strong evidence to support increasing the recommended 25(OH)D target concentration to greater than 50 nmol L^−1^ [284], which is the concentration recommended by many organisations internationally.

#### Revised action spectrum for vitamin D

Action spectra are biological weighting functions that are used to assess the risks and benefits of exposure to different wavelengths of UV radiation. An action spectrum for the production of pre-vitamin D in the skin was produced by the Commission Internationale l’Éclairage (CIE) in 1982, showing a maximum effect at 297 nm, with essentially no production above 315 nm. However, the validity of this has been questioned because it is based on the use of human skin ex vivo. A recently published study calculated the action spectrum for serum 25(OH)D, the accepted molecule to determine vitamin D status, using an in vivo experiment [285]. The action spectrum was shifted 5 nm towards shorter wavelengths, suggesting that the CIE action spectrum may need to be revised. However, the effect of the shift is likely to be less relevant for natural sunlight than for artificial light sources [1]. Thus while further research is needed to elucidate the implications of a revised action spectrum for calculating the ratio of harms *vs* benefits of exposure to sunlight, the CIE action spectrum is likely to be adequate for risk benefit calculations.

#### Effect of clothing, sunscreen, and skin pigmentation on vitamin D production

Clothing provides good protection against erythema but also has a strong inhibitory effect on vitamin D synthesis. Full body clothing cover, especially in females, may contribute to the high prevalence of vitamin D deficiency in many countries with high insolation. Recent studies confirm the influence of clothing on 25(OH)D concentration [286, 287].

Sunscreen reduces the risk of skin cancer and premalignant lesions and is a mainstay of sun protection globally, but concerns have been raised that regular application of sunscreen may increase the risk of vitamin D deficiency. Two reviews suggest this is not the case [288, 289], although there are no RCTs of the effect of routine application of high SPF sunscreen on 25(OH)D concentration. Studies conducted since these reviews continue to suggest that sunscreen users have higher 25(OH)D concentration than those who do not use sunscreen [290, 291]. This is most likely because sunscreen users spend more time outdoors, but these studies suggest that using sunscreen does not obviate the benefits for vitamin D of spending more time outdoors.

Dark-skinned immigrants to northern European countries tend to have lower 25(OH)D concentration than those with lighter skin. This is likely due to a combination of reduced vitamin D production in darker compared with lighter skins, and to behavioural differences. For example, an observational study comparing Danes with dark and light skin found that those with dark skin received a lower UV radiation dose and exposed less body surface area than those with lighter skin. There was only minimal difference in the increase in 25(OH)D concentration per joule of UV radiation exposure (light = 0.63 nmol L^−1^ J^−1^; dark = 0.53 nmol L^−1^ J^−1^) [292], although the analysis assumed a proportional response in 25(OH)D concentration with increasing body surface area exposed, which may not be the case. Experimental studies have generated discrepant estimates of the inhibitory effect of melanin. One study examined the effect on 25(OH)D concentration of exposing people with different skin types to five serial whole-body sub-erythemal exposures of solar-simulated UV radiation [293]. Comparing people with very light and very dark skin, the melanin inhibitory factor was estimated at ~ 1.3. In contrast, in a study in which the dose of solar-simulated radiation was given as a function of minimum erythemal dose (i.e. people with darker skins received a higher dose), and UV radiation was delivered to commonly exposed skin sites only, the melanin inhibitory factor was estimated to be ~ 8 [294]. This issue needs to be resolved as it has implications for public health advice for people with darker skin.

#### Prevalence of vitamin D deficiency

**Fig. 5:**
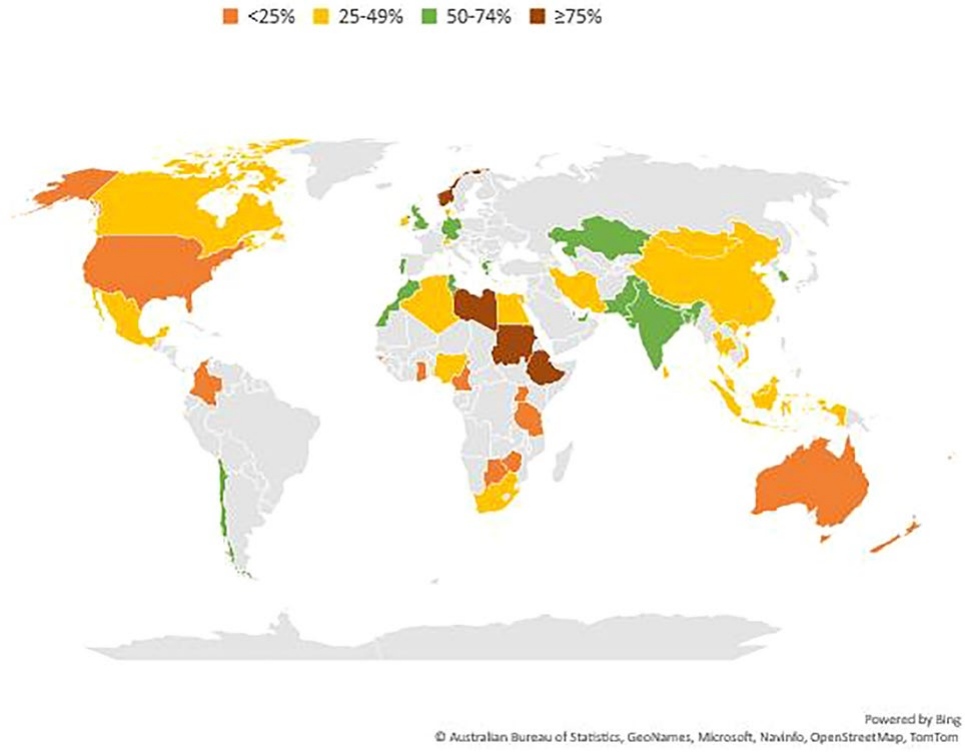
Prevalence of vitamin D deficiency (25(OH)D < 50 nmol/L). Figures for south-Asian countries (Sri Lanka, Nepal, Bangladesh, Pakistan, and India) are derived from a meta-analysis of studies (see Online Resource Table 2) that included a range of different populations and 25(OH)D assays. Similarly, figures for African countries are derived from a meta-analysis of studies (see Online Resource Table 3) that included a range of different populations and 25(OH)D assays. All other figures are based on population surveys. Data for Chile and Fiji are restricted to women. Data for Mongolia are restricted to men. Data for Denmark, Norway, Greece, Mexico, Ireland, and Iran are restricted to children and/or adolescents. For details of the adult age ranges for other countries see Online Resource Table 1

Vitamin D deficiency is prevalent across many parts of the world. However, obtaining accurate estimates is hampered by unreliable laboratory assays used in many studies. The prevalence of deficiency also depends on the 25(OH)D concentration used to define deficiency and on the time of year when samples were collected; these factors must be considered when interpreting these data. Figure 5 (with detailed data in Online Resource Table 1) shows the prevalence of vitamin D deficiency (25(OH)D concentration < 50 nmol L^−1^) derived from national studies (albeit using a range of assays, not all standardised to an international standard reference method) as well as some recent population studies. The results of these prevalence studies emphasise the apparent high prevalence of vitamin D deficiency in many parts of the world. With recovery of stratospheric ozone under the Montreal Protocol, projections are for lower UV-B radiation at high-latitude locations [1], which could increase the prevalence of vitamin D deficiency. This effect may be ameliorated by warming temperatures due to climate change, resulting in greater time outdoors, as demonstrated by a study from Germany, which found significantly higher 25(OH)D concentration in two extreme summers (2018 and 2019) compared with the preceding 4 summers [295]. However, in lower-latitude locations where the temperature is already high, warming temperatures due to climate change may reduce time outdoors and exacerbate the problem of vitamin D deficiency, particularly in urban populations.

### Climate change, depletion of stratospheric ozone, and human health

In a previous assessment [79] we comprehensively reviewed the links between climate change, stratospheric ozone depletion and recovery, and human health. Little has been published on this topic in the last 4 years. The Sixth Assessment of the IPCC Working Group II on the effects of climate change on human health does not mention skin cancer, or other UV-induced health outcomes. Projections for ambient UV radiation in the coming years [1] suggest that with recovery of stratospheric ozone there will be a reduction in the UVI of 2–5% in northern mid-latitudes, a reduction of 4–6% in southern mid-latitudes, and no change in the tropics. Although large reductions in the UV Index in southern high latitudes (> 60°S) as stratospheric ozone recovers are projected, this is in a region with no resident populations (Ushuaia in southern Argentina has latitude of 55°S). However, with a growing number of tourists to Antarctica each summer season (approximately 170,000/season in recent years [296]) as well as base staff and researchers, the high variability in the UV Index, including maxima reaching UV Index of 14 [1] may pose risks to health. In addition to the effects of recovery of stratospheric ozone, reductions in cloud cover are projected to increase DNA-weighted UV radiation levels by 1.3% per decade from 2050, based on data from 1998 to 2016 at four mid-latitude sites (Lauder, New Zealand; Table Mountain, South Africa; Haute Provence, France; Hohenpeissenberg, Germany) and one tropical high altitude site (Mauna Loa, Hawaii) [297].

In an extension of a previous analysis [298], Piacentini and colleagues applied a temperature modification to the carcinogenicity of UV radiation (‘effective carcinogenicity’) to estimate the incidence of KC in current and next centuries as a result of rising ambient temperature under different climate change scenarios (RCP2.6, RCP4.5, RCP6.0, RCP 8.5) [299]. The model projects increases in incidence for SCC for 2100 of 5.8%, 10.4%, 13.8% and 21.4% (for respective RCPs), and for BCC, 2.1%, 4.9%, 6.5% and 9.9%. The model does not take account of changing UV radiation (as a result of changes in stratospheric ozone and/or cloud cover), or changes in sun exposure behaviour in relation to temperature.

## Gaps in knowledge

During our assessment of the literature, we identified the following gaps in knowledge:

- Dynamic modelling is needed to better quantify the benefits of the Montreal Protocol: Trends in skin cancer in different countries are likely to be due to a combination of: (1) immigration patterns, leading to changed distribution of skin types; (2) changing recreational and occupational exposures; (3) concerted efforts to encourage populations to adopt sun-protective behaviours; and (4) changing surveillance habits, potentially resulting in over-diagnosis. Importantly, any predictions will need to take account of the influence of climate change on human behaviour, which will be an increasingly important driver of exposure to UV radiation.
- Better methods are needed to estimate prevalence of health conditions, including keratinocyte cancer, related to UV radiation: Other than internal cancers and melanoma, the lack of population-based registries makes it extremely challenging to estimate the population incidence or prevalence of conditions, including keratinocyte cancers, related to exposure to sunlight. Harnessing the power of data linkage may be one way of resolving this problem, recognising that this may under-estimate the burden of conditions that present less frequently in the health system.
- Photobiological studies to define the dose and pattern of UV radiation that confers minimal harm are required: There is currently no known UV radiation dose or exposure pattern that confers minimal harm to the skin and eyes. Related to this, over-exposure is poorly defined and, in some settings, sunburn is considered the only relevant indicator of over-exposure. A greater understanding of this issue would enable messages to be developed that balance the benefits and harms of exposure to sunlight.
- The extent of the problem relating to the use of photosensitising medications needs to be elucidated: Photosensitising medications can result in damage to skin and eyes. However, the extent of the problem, particularly for the eyes, is unclear.
- Studies are needed to better understand beneficial effects of exposure to UV radiation: Exposing the skin and eyes to the sun is likely to have benefits beyond those mediated by production of vitamin D. However, while evidence of benefit and mechanisms is maturing, it is still in its infancy. Clearly defined non-vitamin D biomarkers of benefit are needed so that studies can be conducted to identify the mechanisms, along with the dose and pattern of exposure needed to confer benefits.
- Public health messaging to guide personal sun exposure to minimise harms and maximise any benefits requires more detail on the relative effective doses of UV radiation: In particular, we need to quantify the effect on the balance of risks and harms of smaller doses of UV radiation to a greater body surface area. For example, a comparison of the effect of exposure to UV radiation with 5% and 85% of the body surface area exposed suggests that there may not be a linear increase in 25(OH)D concentration, but there is little information about percentages between these extremes.
- Current public health messages focus on lightly pigmented populations, with doses of UV radiation for harms and benefits uncertain for deeply pigmented skin: Skin cancers are rare, but vitamin D deficiency common, in those with deeply pigmented skin. Skin melanin protects the skin from UV-B-induced harms (e.g. DNA damage) and reduces vitamin D production, but the UV radiation dose at which these events occur needs to be quantified. Further, the action spectrum for vitamin D production may vary with skin type, but this is not currently known. Resolving these questions is important, enabling the development of evidence-based messages that recognise the increasing diversity of populations within countries.

## Conclusions 

Exposure to UV radiation has multiple harms and benefits. By preventing large increases in UV-B radiation, the Montreal Protocol has avoided many adverse health outcomes, consistent with Sustainable Development Goal (SDG) 3 (Ensure healthy lives and promote well-being for all at all ages). Further, the costs of the adverse effects of exposure to UV radiation are high and increasing, and occupational exposures represent a considerable economic burden. The Montreal Protocol plays a role in protecting outdoor workers, consistent with SDG 8 (Promote sustained, inclusive and sustainable economic growth, full and productive employment and decent work for all).

In addition to avoiding large increases in UV radiation, the Montreal Protocol has stimulated research into the harms and benefits of sunlight exposure. The resulting knowledge has enabled harms to the skin and eyes to be ameliorated through the use of sun protection strategies. For the skin, this has been particularly important for people with lightly pigmented skin, and is evident in the plateauing trends in skin cancer seen in younger age groups in some countries. For the eyes, blindness caused by cataracts disproportionately affects people in developing countries due to lack of access to lens replacement surgery. These diverse effects are consistent with SDG 10 (Reduce inequality within and among countries).

Alongside the harms, increasing recognition of the benefits is informing public health and clinical practice. For people with lightly pigmented skin, this underpins strategies to balance the risks and benefits of sun exposure. For those with deeply pigmented skin, knowledge of the importance of sun exposure may be particularly relevant for those living in areas with low ambient UV radiation for whom the benefits of sun exposure for most people (with the exception of those at risk of inflammatory skin disorders) are likely to outweigh the harms.

In conclusion, sun exposure is critical for human life on Earth. The Montreal Protocol and its Amendments have prevented large increases in ambient UV-B radiation. This has both mitigated the adverse effects and enabled access to the beneficial effects of sun exposure, thus playing a vital role globally in health and economies.

## Supplementary Information

Below is the link to the electronic supplementary material.

Supplementary file1 (DOCX 347 KB)

## Data Availability

Global Cancer Observatory data used in this manuscript are publically available. No other data were used.

## Footnotes

1 25(OH)D is the metabolite measured to determine vitamin D status.

2 Conversion to euros for all values where the original value is another currency performed on 12 October 2022.

3 Fitzpatrick Skin Types: Type 1: Light white skin, always burns, never tans; Type 2: Light white skin, usually burns, tans minimally; Type 3: White skin, sometimes burns, tans moderately; Type 4: Olive-light brown skin, rarely burns, tans moderately heavily; Type 5: Medium brown skin, rarely burns; Type 6: Dark brown–black skin, rarely to never burns.
