# Supplementary material for: The effects of exposure to solar radiation on human health
Source: Photochem Photobiol Sci. 2023 Mar 1:1–37. Online ahead of print. doi: 10.1007/s43630-023-00375-8 (PMC9976694; doi:10.1007/s43630-023-00375-8)
Supplement: Supplementary file 1 — Supplementary file1 (DOCX 347 KB) [file 43630_2023_375_MOESM1_ESM.docx]

Supplementary table 1: Prevalence of vitamin D deficiency from national studies and published peer-reviewed papers

| **Author, year [Publication number]** | **Location** | **Study years** | **Population** | **Assay** | **Season** | **n** | **Age** | **Percent vitamin D deficient (25(OH)D< 50 nmol/L)** |
| --- | --- | --- | --- | --- | --- | --- | --- | --- |
| Australian Bureau of Statistics [1] | Australia | 2011-3 | Random sample | LC-MS/MS | Year round | ~11,000 | 5+ years | 23.5 |
| Black et al 2021 [12] | Australia |  | Nationally representative, Aboriginal and Torres Strait Islanders | LC-MS/MS |  | 3250 | adult | 27.0 |
| New Zealand Ministry of Health [6] | New Zealand | 2008-9 | Nationally representative | LC-MS/MS | Year round | 3099 | 15+ y | 32.0 |
| Yousef et al 2021 [2] | Canada | 2012-5 | Random sampling of dwellings | Diasorin Immunoassay | Year round | 11,579 | 3-79 y | 36.4 |
| Herrick et al 2019 [3] | United States | 2011-4 | NHANES – probability sample | LC-MS/MS | South in winter, north in summer | 16,180 | ≥1 y | 18.3 |
| Cashman et al 2013 [5] | Ireland | 2019-20 | Nationally representative | LC-MS/MS | Year round | 246 | 13-18 y | 54.8 |
|  | Ireland | 2008-10 | Random selection | ELISA (OCTEIA)  LC-MS/MS | Year round | 1132 | 18+ y | 40.1  45.9 |
| Cashman et al 2016 [14] | Denmark | 2011-2 | Regionally representative | Variable, but standardised to the Vitamin D standardisation program |  | 779 | 8-12 y | 36.8 |
|  | Norway | 2010-11 | Regionally representative |  |  | 939 | 15-18 y | 76.1 |
|  | Greece | 2007-9 | Regionally representative |  |  | 806 | 9-14 y | 62.4 |
|  | Germany | 2003-6 | Nationally representative |  |  | 10,015 | 1-17 y | 45.6 |
|  | Germany | 2008-11 | Nationally representative |  |  | 6995 | 18-79 y | 56.0 |
|  | United Kingdom | 2008-12 | Nationally representative |  |  | 511 | 1-18 y | 53.4 |
|  | United Kingdom | 2008-12 | Nationally representative |  |  | 977 | 19-91 y | 57.9 |
|  | Netherlands | 2006-7 | Regionally representative |  |  | 2627 | 40-66 y | 33.6 |
|  | Iceland | 2002-6 | Regionally representative |  |  | 5519 | 66-96 y | 33.6 |
| Lin et al 2021 [15] | United Kingdom | 2006-10 | UK Biobank | Diasorin Liaison | Year round | 449,943 | 40-69 y | 32.3 |
| Kim et al 2020 [4] | South Korea | 2008-14 | Randomly selected households | Diasorin RIA | Year round | 46,405 | 18-80 y | 66.7 |
| Chile Ministry of Health [7] | Chile | 2016-7 | Nationally representative | LC-MS/MS |  | 5520 | 15-49 y females | 54.8 |
| Mongolia National Center for Public Health [8] | Mongolia | 2016-7 | Nationally representative | Enzyme-linked fluorescence assay | Sept-Nov | 1711 | 6-59 m | 61.0 |
|  |  |  |  |  | Sept-Nov | 377 | 15-49 y men | 40.4 |
|  |  |  |  |  | Sept-Nov | 924 | Pregnant women | 75.4 |
| Heere et al 2010 [9] | Fiji | 2004 | Nationally representative | Diasorin RIA | Winter (May-Sept) | 511 | 15-54 y females | 11.0 |
| Marzban et al 2021 [10] | Iran |  | Randomly selected households living in rural areas of the Persian Gulf | IDS-ELISA |  | 1806 | Adults | 28.0 |
| Duarte et al 2020 [11] | Portugal |  | Nationwide, random sample of people living in private households | ADVIA competitive immunoassay |  | 3092 | 18+ y | 67.0 |
| Beer et al, 2020 [13] | Colombia |  | National survey | ADVIA competitive immunoassay |  | ~40,000 | All ages | 24.0 |
|  |  |  |  |  |  |  | Toddlers | 43.0 |
|  |  |  |  |  |  |  | Adolescents | 20.0 |
| Flores et al 2017 28241892 | Mexico | 2012 | Nationally representative | Chemiluminescent microparticle immunoassay | Year round | 2695 | 1-4 y | 25.9 |
|  |  |  |  |  |  |  | 5-11y | 36.6 |
| Gromova et al 2020 [16] | Kazakhstan | 2018 | Systematic random sampling from outpatient clinics in each region | Abbott chemilumi-  nescence microparticle immunoassay (CMIA) | Summer (May to Aug) | 1347 | Adults females | 79.0 |
|  |  |  |  |  |  |  | Adult males | 56.1 |
| Chen et al 2017 [17] | China | 2010-2013 | Nationally representative | Diasorin RIA | Year round | 6014 | 60+ y | 39.1 |
| Hu et al 2017 [18] | China | 2010-2013 | Nationally representative | Diasorin RIA | Year round | 14,473 | 6-17 y | 53.2 |
| Nikooyeh et al 2021 [19] | Iran | 2013-4 | Nationally representative | Direct enzyme Immunoassay | Year round | 1111 | 19-65 y | Winter: 90.1  Summer: 69.2 |
| Nikooyeh et al 2017 [20] | Iran | 2013-4 | Nationally representative | Direct enzyme Immunoassay | Year round | 667 | 5-18 y | Mid-Winter: 93.2 |
| Poh et al 2013 [21] | Malaysia | 2010-1 | Nationally representative | Diasorin Liaison | Year round | 2056 | 4-12 y | 47.5 |
| Poh et al 2016 [22] | Malaysia | 2010-1 | Nationally representative | Diasorin Liaison | Year round | 861 | 2+ years | 43.7 |
|  | Thailand |  |  | Diasorin Liaison |  | 495 |  | 33.7 |
|  | Indonesia |  |  | Immunoassay |  | 276 |  | 44.0 |
|  | Vietnam |  |  | HPLC |  | 384 |  | 48.2 |
| Bani et al 2022 [23] | Switzerland, Canton Ticino |  |  | Chemiluminescence microparticle capture immunoassay (CMIA) |  | 18,131 | All | 35.5 |
| Al Zarooni 2019 [24] | Abu Dhabi Emirates | 2011-2 | Adults presenting to preventive health screening program clinics | Not provided | Year round | 12,346 | 18+ years | 72.0 |
| Latif Zainel et al 2019 [25] | Qatar | 2017 | Adults presenting to primary care clinics | Abbott immunoassay; multiple labs | Year round | 102,342 | 18-65 y | Not treated: 71.4  Previously treated: 53.4 |

Values in red show those selected for the map displayed in Figure 5.

Supplementary Table 2: Prevalence of vitamin D deficiency in South Asian countries (results from a meta-analysis) [26]

| **Country** | **Percent vitamin D deficient**  **(25(OH)D< 50 nmol/L)** | Heterogeneity | No. studies |
| --- | --- | --- | --- |
| Sri Lanka | 48 |  | 1 |
| Nepal | 57 |  | 2 |
| Bangladesh | 67 |  | 5 |
| Pakistan | 73 | High (18% to 99%) | 18 |
| India | 67 | High (7% to 91%) | 39 |

Supplementary table 3: Prevalence of vitamin D deficiency in African countries (results from a meta-analysis) [27]

| **Country** | **Percent vitamin D deficient (25(OH)D< 50 nmol/L)** | **Population** | No. studies |
| --- | --- | --- | --- |
| Tunisia (2) | 68 | Adults | 2 |
| Libya (1) | 80 | Adults | 1 |
| Egypt (5) | 45 | Adults | 5 |
| Sudan (1) | 99 | Adults | 1 |
| Ethiopia (4) | 86 | Adults | 4 |
| Uganda (1) | 20 | Adults | 1 |
| Kenya (1) | 17 | Adults | 1 |
| Seychelles (1) | 8 | Adults | 1 |
| Tanzania (3) | 3 | Adults | 3 |
| Zimbabwe (1) | 2 | 1-2 year olds (but said to be similar to adult levels) | 1 |
| South Africa (7) | 31 | Adults | 7 |
| Botswana (1) | 17 | <2yo without tuberculosis | 1 |
| Cameroon (1) | 3.2 | Adults | 1 |
| Nigeria (5) | 25 | Adults | 5 |
| Ghana (3) | 14 | Adults | 3 |
| Cote d’Ivoire (1) | 9 | Adults | 1 |
| Guinea-Bissau (1) | 13 | Adults | 1 |
| Morocco (2) | 74 | Adults | 2 |
| Algeria (1) | 29.9 September  41.4 March | 5-15 yo children | 1 |

Supplementary Figure 1: UV radiation-induced signature mutations in DNA

DNA contains 4 amino acid bases (thymine (T), adenine (A), guanine (G), cytosine (C)), linked by a sugar phosphate backbone. DNA is in the form of a double helix with two anti-parallel strands, one running in the 5’ (5-prime) to 3’ direction, and the other in the 3’ to 5’ direction (5’ and 3’ refer to carbon molecules in the sugar backbone). Thymine and cytosine are pyrimidines; guanine and adenine are purines. Thymine binds (with a weak hydrogen bond) only to adenine, cytosine to only guanine. DNA is ‘read’ to construct proteins – the correct sequence of bases is critical to ensure the correct structure of the proteins synthesised.


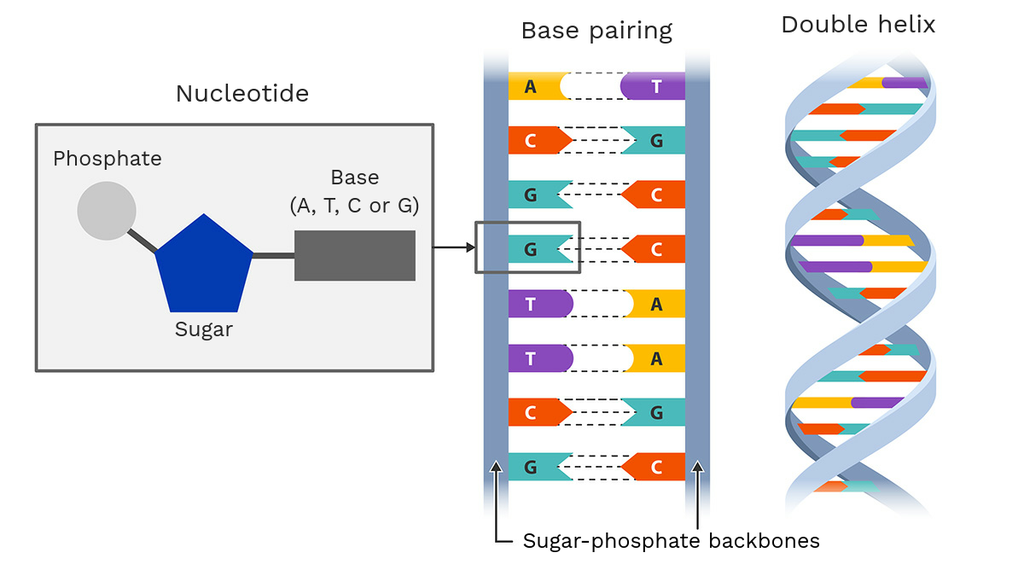


3’

5’

3’

5’

Nucleotides are composed of a sugar molecule, a phosphate group, and a base. TpC refers to two adjacent nucleotides, where a thymine nucleotide is followed by a cytosine nucleotide in the sequence going from the 5’ to the 3’ direction (for example in the red circle above), and similarly, CpC describes a cytosine followed by a cytosine in the 5’ to 3’ direction.

Absorption of UV-B radiation by DNA can cause adjacent thymine bases to ‘join up’ to form a thymine dimer (a form of cyclobutane pyrimidine dimer (CPD)).


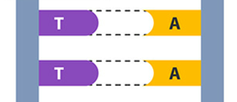

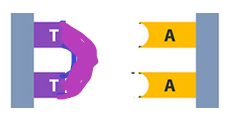


If thymine dimers are not repaired, the result can be death of the cell. Repair enzymes can locate the CPD, remove it, and use the other DNA strand as a guide to repair the DNA correctly. However, faulty DNA repair can lead to the original cytosine (C) base being replaced by a thymine base (T), resulting in a permanent change in the sequence of bases for that strand of DNA; i.e., a UV signature mutation. Mutations result in incorrect “reading” of DNA and production of abnormal proteins.

- a C>T transition at a TpC dinucleotide (see red circled area above) due to faulty DNA repair of a T-T dimer.


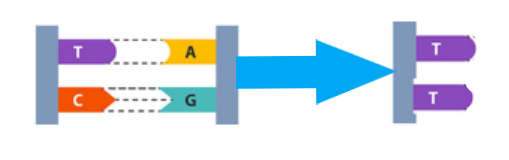


- a C>T transition at a CpC dinucleotide (either the first or second cytosine replaced, CpC and CpC respectively) due to faulty DNA repair of UV-induced DNA damage


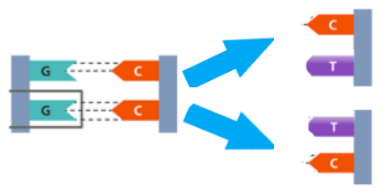


REFERENCES

1. Australian Bureau of Statistics. The Australian Health Survey 2011-2013: Updated results. .

2. Yousef, S., Manuel, D., Colman, I., Papadimitropoulos, M., Hossain, A., Faris, M., & Wells, G. A. (2021). Vitamin D Status among First-Generation Immigrants from Different Ethnic Groups and Origins: An Observational Study Using the Canadian Health Measures Survey. *Nutrients, 13*(8), <https://doi.org/10.3390/nu13082702>

3. Herrick, K. A., Storandt, R. J., Afful, J., Pfeiffer, C. M., Schleicher, R. L., Gahche, J. J., & Potischman, N. (2019). Vitamin D status in the United States, 2011-2014. *American Journal of Clinical Nutrition, 110*(1), 150-157, <https://doi.org/10.1093/ajcn/nqz037>

4. Kim, S. Y., Lee, M. H., Lim, W. J., Kim, S. I., & Lee, Y. J. (2020). Associations of 25-Hydroxyvitamin D Levels and Arthritis with Sleep Duration: The Korean National Health and Nutrition Examination Survey 2008-2014. *Nat Sci Sleep, 12*, 883-894, <https://doi.org/10.2147/NSS.S275464>

5. Cashman, K. D., Muldowney, S., McNulty, B., Nugent, A., FitzGerald, A. P., Kiely, M., Walton, J., Gibney, M. J., & Flynn, A. (2013). Vitamin D status of Irish adults: findings from the National Adult Nutrition Survey. *British Journal of Nutrition, 109*(7), 1248-1256, <https://doi.org/10.1017/S0007114512003212>

6. Ministry of Health (2012). Vitamin D status of New Zealand Adults: Findings from the 2008/09 New Zealand Adult Nutrition Survey. Wellington: Ministry of Health.

7. Chile Ministry of Health (2017). National Health Survey 2016/2017. <http://epi.minsal.cl/wp-content/uploads/2018/03/Resultados-Vitamina-D.pdf>. Accessed 11 October 2022.

8. National Center for Public Health (2017). Nutrition status of the population of Mongolia: Fifth National Health Survey report

9. Heere, C., Skeaff, C. M., Waqatakirewa, L., Vatucawaqa, P., Khan, A. N., & Green, T. J. (2010). Serum 25-hydroxyvitamin D concentration of Indigenous-Fijian and Fijian-Indian women. *Asia Pac J Clin Nutr, 19*(1), 43-48,

10. Marzban, M., Kalantarhormozi, M., Mahmudpour, M., Ostovar, A., Keshmiri, S., Darabi, A. H., Khajeian, A., Bolkheir, A., Amini, A., & Nabipour, I. (2021). Prevalence of vitamin D deficiency and its associated risk factors among rural population of the northern part of the Persian Gulf. *BMC Endocrine Disorders, 21*(1), 219, <https://doi.org/10.1186/s12902-021-00877-5>

11. Duarte, C., Carvalheiro, H., Rodrigues, A. M., Dias, S. S., Marques, A., Santiago, T., Canhão, H., Branco, J. C., & da Silva, J. A. P. (2020). Prevalence of vitamin D deficiency and its predictors in the Portuguese population: a nationwide population-based study. *Archives of Osteoporosis, 15*(1), 36-36, <https://doi.org/10.1007/s11657-020-0695-x>

12. Black, L. J., Dunlop, E., Lucas, R. M., Pearson, G., Farrant, B., & Shepherd, C. C. J. (2021). Prevalence and predictors of vitamin D deficiency in a nationally representative sample of Australian Aboriginal and Torres Strait Islander adults. *British Journal of Nutrition, 126*(1), 101-109, <https://doi.org/10.1017/S0007114520003931>

13. Beer, R. J., Herran, O. F., & Villamor, E. (2020). Prevalence and correlates of vitamin D deficiency in a tropical setting: results from a nationally representative survey. *American Journal of Clinical Nutrition, 112*(4), 1088-1098, <https://doi.org/10.1093/ajcn/nqaa197>

14. Cashman, K. D., Dowling, K. G., Skrabakova, Z., Gonzalez-Gross, M., Valtuena, J., De Henauw, S., Moreno, L., Damsgaard, C. T., Michaelsen, K. F., Molgaard, C., Jorde, R., Grimnes, G., Moschonis, G., Mavrogianni, C., Manios, Y., Thamm, M., Mensink, G. B., Rabenberg, M., Busch, M. A., Cox, L., Meadows, S., Goldberg, G., Prentice, A., Dekker, J. M., Nijpels, G., Pilz, S., Swart, K. M., van Schoor, N. M., Lips, P., Eiriksdottir, G., Gudnason, V., Cotch, M. F., Koskinen, S., Lamberg-Allardt, C., Durazo-Arvizu, R. A., Sempos, C. T., & Kiely, M. (2016). Vitamin D deficiency in Europe: pandemic? *American Journal of Clinical Nutrition, 103*(4), 1033-1044, <https://doi.org/10.3945/ajcn.115.120873>

15. Lin, L. Y., Smeeth, L., Langan, S., & Warren-Gash, C. (2021). Distribution of vitamin D status in the UK: a cross-sectional analysis of UK Biobank. *BMJ Open, 11*(1), e038503, <https://doi.org/10.1136/bmjopen-2020-038503>

16. Gromova, O., Doschanova, A., Lokshin, V., Tuletova, A., Grebennikova, G., Daniyarova, L., Kaishibayeva, G., Nurpeissov, T., Khan, V., Semenova, Y., Chibisova, A., Suzdalskaya, N., Aitaly, Z., & Glushkova, N. (2020). Vitamin D deficiency in Kazakhstan: Cross-Sectional study. *Journal of Steroid Biochemistry and Molecular Biology, 199*, 105565, <https://doi.org/10.1016/j.jsbmb.2019.105565>

17. Chen, J., Yun, C., He, Y., Piao, J., Yang, L., & Yang, X. (2017). Vitamin D status among the elderly Chinese population: a cross-sectional analysis of the 2010-2013 China national nutrition and health survey (CNNHS). *Nutr J, 16*(1), 3, <https://doi.org/10.1186/s12937-016-0224-3>

18. Hu, Y., Chen, J., Wang, R., Li, M., Yun, C., Li, W., Yang, Y., Piao, J., Yang, X., & Yang, L. (2017). Vitamin D Nutritional Status and its Related Factors for Chinese Children and Adolescents in 2010-2012. *Nutrients, 9*(9), <https://doi.org/10.3390/nu9091024>

19. Nikooyeh, B., Abdollahi, Z., Shariatzadeh, N., Kalayi, A., Zahedirad, M., & Neyestani, T. (2021). Effect of latitude on seasonal variations of vitamin D and some cardiometabolic risk factors: national food and nutrition surveillance. *East Mediterr Health J, 27*(3), 269-278, <https://doi.org/10.26719/emhj.20.119>

20. Nikooyeh, B., Abdollahi, Z., Hajifaraji, M., Alavi-Majd, H., Salehi, F., Yarparvar, A. H., & Neyestani, T. R. (2017). Vitamin D Status, Latitude and their Associations with Some Health Parameters in Children: National Food and Nutrition Surveillance. *J Trop Pediatr, 63*(1), 57-64, <https://doi.org/10.1093/tropej/fmw057>

21. Poh, B. K., Ng, B. K., Siti Haslinda, M. D., Nik Shanita, S., Wong, J. E., Budin, S. B., Ruzita, A. T., Ng, L. O., Khouw, I., & Norimah, A. K. (2013). Nutritional status and dietary intakes of children aged 6 months to 12 years: findings of the Nutrition Survey of Malaysian Children (SEANUTS Malaysia). *British Journal of Nutrition, 110 Suppl 3*, S21-35, <https://doi.org/10.1017/S0007114513002092>

22. Poh, B. K., Rojroongwasinkul, N., Nguyen, B. K., Sandjaja, Ruzita, A. T., Yamborisut, U., Hong, T. N., Ernawati, F., Deurenberg, P., Parikh, P., & Group, S. S. (2016). 25-hydroxy-vitamin D demography and the risk of vitamin D insufficiency in the South East Asian Nutrition Surveys (SEANUTS). *Asia Pac J Clin Nutr, 25*(3), 538-548, <https://doi.org/10.6133/apjcn.092015.02>

23. Bani, P., Cingari, S., & Mathis, B. (2022). Study on the prevalence of vitamin D deficiency in the population of Canton Ticino. *La Rivista Italiana della Medicina di Laboratorio, 18*(1), 18-21, <https://doi.org/10.23736/S1825-859X.22.00136-0>

24. Al Zarooni, A. A. R., Al Marzouqi, F. I., Al Darmaki, S. H., Prinsloo, E. A. M., & Nagelkerke, N. (2019). Prevalence of vitamin D deficiency and associated comorbidities among Abu Dhabi Emirates population. *BMC Res Notes, 12*(1), 503, <https://doi.org/10.1186/s13104-019-4536-1>

25. Zainel, A. A. L., Qotba, H., Al Nuaimi, A., & Syed, M. (2019). Vitamin D status among adults (18-65 years old) attending primary healthcare centres in Qatar: a cross-sectional analysis of the Electronic Medical Records for the year 2017. *BMJ Open, 9*(8), e029334, <https://doi.org/10.1136/bmjopen-2019-029334>

26. Siddiqee, M. H., Bhattacharjee, B., Siddiqi, U. R., & MeshbahurRahman, M. (2021). High prevalence of vitamin D deficiency among the South Asian adults: a systematic review and meta-analysis. *BMC Public Health, 21*(1), 1823, <https://doi.org/10.1186/s12889-021-11888-1>

27. Mogire, R., Mutua, A., Kimita, W., Kamau, A., Bejon, P., Pettifor, J., Adeyemo, A., Williams, T., & Atkinson, S. (2020). Prevalence of vitamin D deficiency in Africa: a systematic review and meta-analysis. *The Lancet Global Health, 8*(1), e134-e142,
